# Eco-friendly control of fusarium wilt in tomato: molecular docking and functional analysis of ZnO nanoparticles and biostimulants

**DOI:** 10.3389/fpls.2025.1687653

**Published:** 2025-12-09

**Authors:** Yasmin M. Heikal, Amal M. Albahi, Hala M. Abdelmigid, Amal A. Alyamani, Naglaa Elshafey, Hoda M. Soliman, Samia A. Haroun

**Affiliations:** 1 Botany Department, Faculty of Science, Mansoura University, Mansoura, Egypt; 2 Department of Biotechnology, College of Science, Taif University, Taif, Saudi Arabia; 3 Botany and Microbiology Department, Faculty of Science, Arish University, Al-Arish, Egypt

**Keywords:** *Solanum lycopersicum*, humic acid, salicylic acid, molecular docking, nanoparticles, *fusarium oxysporum*

## Abstract

In *Solanum lycopersicum* L. plant, *Fusarium oxysporum* is a fungal pathogen that leads to heavy tomato losses and severe wilting (FW). This investigation was planned to control *F. oxysporum* through some resistance inducers including salicylic acid (SA)(0, 0.4, 0.5, and 0.6 mM), humic acid (HA)(0, 50, 100, and 150 mg/L), zinc nanoparticles (ZnO-NPs) (0, 100, 250, and 500 mg/L) applications on 35 days- old of tomato plantlets and compared to the negative and positive controls. The selected Fusarium strain was identified at the molecular level using 18S rRNA analysis and assigned with accession number FO-A1 (OM321440). Their antifungal efficacy was demonstrated by the inhibition of *F. oxysporum* growth *in vitro*, improved plant growth under greenhouse conditions *in vivo*, increased antioxidant activity (TAC), total protein and carbohydrate levels, and phenolic content (TPC) inflicted by the fungus. The most effective treatment was 500 mg/L of ZnO-NPs, which reduced disease incidence to 65% and disease severity to 1.0, compared to the inoculated control. In addition to *in silico* investigations of biostimulants and ZnO-NPs against lanosterol 14-alpha-demethylase (CYP51), the principal target of antifungal agents, the binding affinities were determined to be -5.0 and -2.4 Kcal/mol for salicylic acid and humic acid, respectively. ZnO-NPs exhibited metal interaction with the amino acid residues of CYP51, with a binding energy of -2.19 Kcal/mol, indicating the inhibitory potential of biostimulants and ZnO-NPs. By integrating these state-of-the-art methods, this study enhances the existing body of knowledge in the field, addressing crucial gaps in sustainable disease management and offering alternatives to conventional approaches.

## 1 Introduction

Globally, tomato (*Solanum lycopersicum* L.) is one of the most extensively cultivated vegetables, widely recognized as a popular and commercially significant horticultural crop (Srinivas et al., 2019; Jamil et al., 2021). Due to its high content of vitamins C and A, it constitutes an essential component of daily nutrition and is consumed in various forms of processed fruit products and in its fresh, unprocessed state (Brookie et al., 2018). *Fusarium* species are known to cause a diverse range of disorders in an extensive variety of host plants. *Fusarium oxysporum* is a filamentous fungus belonging to the class Ascomycetes and the family Hypocreaceae, and it is a soil-dwelling fungus that is ubiquitous worldwide. The widespread distribution of *F. oxysporum* in soils globally has led to its inclusion in what is referred to as the *Fusarium oxysporum* species complex (FOSC). *Fusarium* species are typically identified based on their micro- and macroscopic characteristics (Singha et al., 2016). However, phytopathogenic strains cause severe vascular diseases and frequently limit the production of economically significant crops (Servin et al., 2015; Shahzad et al., 2017). As soil-borne pathogens, *F. oxysporum* can persist extensively in the soil as dormant chlamydospores. The presence of the host root induces the germination of chlamydospores. Infection hyphae adhere to the root surface and subsequently penetrate it. The fungus invades the intercellular root cortical cells and enters the vascular system by penetrating the xylem. Subsequently, the pathogen exhibits a specific infection route as it tends to exclusively colonize within xylem vessels, resulting in more rapid colonization of the host. Within vessels, the pathogen begins to produce microconidia, which, upon detachment, are transported upward by the sap stream. Furthermore, in the upper vessels, microconidia germination facilitates fungal penetration (McGovern, 2015).

The characteristic symptoms of wilt are caused by vascular occlusion resulting from the aggregation of hyphae and host-pathogen interactions, including the release of toxins, gingiva, telose formation, and gels. Typical disease markers, such as defoliation, wilting, vein removal, and leaf epilation, appear and eventually precede the death of the host plant. At this stage, vascular wilt fungus, confined to the xylem vessels, spreads among the parenchymal tissue and commences prolific growth on the plant’s surface, including leaves, stems, and other structures (Joshi, 2018). Fusarium wilt (FW) poses a significant threat to tomato production, making its control essential for maintaining plant vigor and fruit quality and quantity. FW presents a considerable challenge in terms of control, although numerous strategies have been proposed to manage and mitigate the spread of this lethal pathogen (Panno et al., 2021).

Current methods employed in the control of FW encompass cultural, biological, chemical, and natural product approaches. Agricultural chemicals, fungicides frequently utilized for pest and disease management include methyl bromide, chloropicrin, and metam sodium for soil disinfection. However, these chemicals are costly and environmentally unfavorable (Singh et al., 2017). Field sanitation, currently considered the most effective control method, involves cleaning and sterilizing agricultural implements before use in unaffected fields. Cultural practices, such as the complete removal and destruction of infected plants, represent another potential approach to controlling the fungal pathogen (Joshi, 2018). Additionally, crop rotation with a non-host crop for more than four seasons has demonstrated positive outcomes in controlling *F. oxysporum* (Sampaio et al., 2020). Soil solarization and sterilization are other methods employed to control Fusarium. However, soil sanitization through fungicides faces challenges due to the existence of asymptomatic Fusarium species (Joshi, 2018).

Resistance inducers, which can be synthetic compounds, plant or microbe components, or Microbial or Pathogen Associated Molecular Patterns (MAMPs or PAMPs), cause a resistance response in plants, resulting in PAMP Triggered Immunity (PTI) (Alexandersson et al., 2016). These inducers, such as Jasmonic Acid (JA), Salicylic Acid (SA), and Ethylene (ET), activate signaling via hormonal pathways and induce a variety of biochemical and molecular responses, including calcium concentration changes, G-protein activation, ubiquitin-dependent protein degradation, and Mitogen-Activated Protein Kinase (MAPK) phosphorylation.

Salicylic acid (SA) represents a promising approach in controlling fungal and bacterial diseases within an environmentally friendly integrated system (Subban et al., 2019). SA, a natural phenolic compound derived from white willow (*Salix alba*), influences various biochemical and molecular processes associated with disease resistance induction. Phenolic compounds exhibit fungitoxic, antibacterial, and antiviral properties (Husien and Yousif, 2018). SA has been effectively utilized to control several plant diseases, including root rot in cucumber, wheat, and tomato, as well as root rot/wilt in sesame (Mohamed and Amer, 2014). Humic acid (HA) solutions produced from potassium humates have shown effectiveness in a variety of plant production applications, including as a plant growth stimulant and soil conditioner. These treatments improve natural plant disease resistance (Abd El-Hai et al., 2011), promote plant growth via accelerated cell division, optimize nutrient and water intake, and motivate soil microbes (Chen and Yada, 2011). Multiple studies have reported the effectiveness of HA in mitigating certain plant diseases (El-Mohamedy et al., 2017). Additionally, Loffredo, Berloco (Loffredo et al., 2007) found that humic acid compounds dramatically decreased radial development and spore germination of *Fusarium oxysporum* f. sp. *lycopersici* and *Fusarium oxysporum* f. sp. *Melonis.*

Strategies based on “nanoparticle-technology” have yielded significant advancements due to their inherent properties (Sharma et al., 2012). Nanoscale materials, owing to their distinctive characteristics, such as enhanced efficacy, reduced ecotoxicity, and decreased input requirements, present a promising alternative for crop protection that offers numerous advantages over conventional approaches and products (Fu et al., 2019). Metal nanoparticles, such as silica (Si), silver (Ag), aluminium (Al), gold (Au), and zinc (Zn), as well as metal oxides like zinc oxide (ZnO) and titanium dioxide (TiO_2_), are being developed and used for crop pest management (Kannan et al., 2020; Chaturvedi et al., 2022). Among the most widely utilized nano products in numerous industrial applications are both ZnO and its nanoparticles (ZnO-NPs). ZnO has recently been employed as an antibacterial agent in micro- and nanoscale formulations; these nanoparticles could be utilized in agriculture as a plant protectant against diseases and in fertilizer products as micronutrients (Gutiérrez-Miceli et al., 2021). In recent years, nanoparticles have gained prominence in combating soil-borne fungal diseases due to their distinctive physical and chemical characteristics, as well as their capacity for targeted delivery. Zinc oxide nanoparticles (ZnO NPs) can enhance agricultural productivity (Panwar et al., 2025). Consequently, the application of nanomaterials for managing fungal diseases in plants is considered a viable alternative to synthetic fungicides.

The molecular docking approach has become an integral part of *in-silico* drug research, which involves predicting the interactions of small molecules with proteins at an atomic level (Sahoo et al., 2022). Molecular docking is a computational technique used to predict the preferred orientation and binding affinity of a small molecule (ligand) when interacting with a target macromolecule, typically a protein. This method plays a central role in structure-based drug design, enabling researchers to simulate and analyze the molecular interactions that underlie biological activity (Agu et al., 2023).

This study aims to establish innovative and sustainable methods for managing Fusarium wilt in tomato plants, prioritizing environmentally friendly and health-promoting solutions. It emphasizes the antifungal potential of zinc oxide nanoparticles (ZnO-NPs), salicylic acid (SA), and humic acid (HA) as eco-safe agents against *Fusarium oxysporum*. Additionally, the research incorporates molecular docking analyses alongside experimental evaluations to investigate the interactions between biostimulants and ZnO-NPs with key pathogenic proteins of *Fusarium*. By examining their binding affinities and inhibitory effects, the study enhances our understanding of the molecular mechanisms underlying their antifungal activity.

## 2 Materials and methods

### 2.1 Materials

Thirty-five-day-old seedlings of *Solanum lycopersicum* L. (Hybrid K 186 cultivar) were acquired from the Agriculture Research Centre (ARC), Giza, Egypt, ensuring uniformity in size and shape. Chemicals used in the study were sourced from Sigma Chemical Company, while others of analytical grade were obtained from various local suppliers.

### 2.2 Isolation of *Fusarium* sp.

Infected tomato plants with wilt symptoms were collected from different Egyptian regions. Infected roots were washed, cut into small segments, sterilized with 0.5% sodium hypochlorite for 1 min., rinsed with distilled water, and dried between sterilized filter papers. These samples were placed on PDA medium and incubated at 28 °C for 3 days. Fungal growth from these samples was analyzed. The isolated fungi were purified. A small hyphal tip (1–2 mm) from the colony margin was carefully excised using a sterile needle and transferred to a fresh PDA plate to initiate a pure culture. To ensure purity, the isolates were subcultured two to three times on PDA. The isolates were stored on PDA slants and maintained at 4 °C for further analysis (Dhingra and Sinclair, 1995).

### 2.3 Molecular identification (18S rRNA)

Specific primers for *Fusarium* sp. were designed using Primer 3 software based on the conserved 18S rRNA gene (Accession No. GQ131884.1) (Rozen and Skaletsky, 2000). Forward (GCCAGAGGACCCCTAAACTC) and reverse (CATTTTGCTGCGTTCTTCAT) primers, synthesized by BIONEER Inc., USA, produced a 118 bp PCR product. Genomic DNA was extracted from mycelia using a modified method by Hussain et al. (2012) and stored at -20 °C. PCR amplification was performed using an Eppendorf Mastercycler with 35 cycles (94 °C for 5 min, 30 sec at 94 °C, 54 °C, and 72 °C, followed by a 5-min extension at 72 °C). Products were resolved on a 2% agarose gel with ethidium bromide and visualized using a gel documentation system (microDoc, Cleaver Scientific Ltd., UK). Edited sequence data were aligned using Finch software (v1.4.0), and homology was assessed via BLAST (NCBI). Phylogenetic analysis was conducted using the neighbor-joining method (Senthilkumar et al., 2011), and the sequence data were submitted to NCBI Gene Bank.

### 2.4 Chemical control of *F. oxysporum* in wilted tomato plants

#### 2.4.1 Chemical control using zinc nanoparticles and growth regulators

Salicylic acid (SA) and humic acid (HA) were sourced from El-Gomohoria Company, Mansoura, Egypt. The study employed SA at concentrations of 0, 0.4, 0.5, and 0.6 mM, along with HA at levels of 0, 50, 100, and 150 mg/L, to assess their efficacy in combating wilt disease in tomato plants.

#### 2.4.2 Chemical control using ZnO-NPs and their characterization

Zinc oxide nanoparticles (ZnO-NPs) powder was obtained from Sigma Aldrich, (https://www.sigmaaldrich.com/), and utilized in different concentrations of 0, 100, 250, and 500 mg/L. The characterization was conducted using UV–Visible Spectroscopy by ZnO-NPs were dispersed in sterile deionized water, and spectrum scans ranging from 200 to 700 nm were performed using the UV-3092 Spectrophotometer (Labindia Analytical Instruments) (Jamdagni et al., 2018). The Fourier Transform Infrared Spectroscopy (FTIR) of dried ZnO-NPs was acquired using a Perkin Elmer Frontier Spectrophotometer, employing the Attenuated Total Reflectance (ATR) method (4000–400 cm^-^¹) to detect phytochemical components (Thermo Fisher Scientific FT-IR) (Mathin et al., 2021). To characterize the size and shape of the particles, a suspension of ZnO-NPs was dried on Gilder G200 Transmission Electron Microscopy (TEM) grids and examined using a JEOL JEM 2100 (HRTEM) operating at 200 kV (Arora et al., 2014).

### 2.5 Microbiological studies

#### 2.5.1 Detection of antifungal activity using ZnO-NPs and biostimulants

The antifungal properties of ZnO-NPs against *F. oxysporum* were evaluated using potato dextrose agar (PDA) as the culture medium. Control plates and those treated with ZnO-NPs at 0, 100, 250, and 500 mg/L, SA at 0, 0.4, 0.5, and 0.6 mM, and HA at 0, 50, 100, and 150 mg/L were prepared. After autoclaving, the PDA medium was cooled, and the treatments were incorporated in a laminar flow chamber with continuous stirring for even distribution. The medium was poured into 9 cm Petri dishes. A 5 mm agar disc with 9-day-old fungal mycelia of *F. oxysporum* was placed on the treated media, and the plates were incubated at 25 °C. Mycelial growth was monitored daily along two axes over a 9-day incubation period (González-Merino et al., 2021). All experiments were conducted in triplicate, and fungal colony diameters were recorded in centimeters. The percentage inhibition of radial mycelial growth was calculated as compared to the untreated control using the formula described by Sundar et al (Sundar et al., 1995):


$$\%\;Inhibition\;=\frac{X-Y}{X}\;\;\times 100$$

Where, X= Growth of control plate, Y= Growth of antifungal treated plate.

#### 2.5.2 Fusarium wilt disease assessment in tomato

The assessment of Fusarium wilt disease was conducted 36 days post-inoculation. Disease incidence was quantified as Percent Disease Incidence (PDI) following the method described by Song et al (Song et al., 2004), using the formula:


$$\text{PDI}=\frac{\text{Number of infected plants}}{\text{Total number of plants}}\times 100$$

Disease severity was evaluated according to the 0–3 rating scale proposed by Kobayashi et al (Kobayashi et al., 1997), based on visible symptoms of leaf yellowing, browning, and structural damage to branches and leaves:

0 = No visible symptoms; 1 = Slight yellowing of lower leaves; 2 = Moderate yellowing and browning of leaves, slight wilting, and 3 = Severe wilting, extensive browning, and damage to branches and leaves.

The severity scores were subsequently used to calculate the Disease Severity Index (DSI) for each treatment group, providing a comprehensive measure of disease progression.

### 2.6 *In silico* molecular docking

#### 2.6.1 Ligand preparation

The three-dimensional structures of biostimulants salicylic acid (SA), humic acid (HA), and Voriconazole (antifungal drug against *Fusarium*) were acquired from the PubChem database available on the NCBI website (https://pubchem.ncbi.nlm.nih.gov, accessed on 1 August 2025). Subsequently, energy minimization was conducted utilizing Avogadro software (Hanwell et al., 2012). Besides energy minimization, Avogadro software enables the building of chemical structures, structural optimization, molecular analysis, and visualization.

##### 2.6.1.1 Preparation of ZnO NPS as a simple model

A very simple, minimum cluster model of the ZnO NPS unit was made to do an early evaluation of possible interaction locations. This model does not depict a bulk nanoparticle structure; instead, it illustrates the fundamental chemical moiety. The model comprises two atoms Zinc (Zn^+^) atom chemically bonded to an Oxygen (O^-^) atom and forming Zinc Oxide (ZnO). Force Fields and Electric Charges were assigned according to UFF parameters, yielding a charge of Zn and O atoms. This structure was utilized as a rigid ligand for docking purposes. We are clear that this simple two-atom model makes the complicated electrical, surface, and hydration features of real ZnO nanoparticles much easier to understand. Subsequently, energy minimization was conducted utilizing Avogadro software (Hanwell et al., 2012). Besides energy minimization, Avogadro software enables the building of chemical structures, structural optimization, molecular analysis, and visualization.

#### 2.6.2 Protein preparation and identification of active sites

The alpha-Fold structure of Lanosterol 14-alpha-demethylase from *Fusarium oxysporum* with ID: A0A0J9VU66 (Jumper et al., 2021). The ligand and water molecules were eliminated from the protein prior to docking, and an inadequate number of hydrogen atoms were later introduced. Kollman charges were assigned to each protein atom, and the degrees of freedom for rotatable bonds were assessed using Auto-Dock Tools-1.5.7. A grid box of 40 Å × 40 Å × 40 Å was created for biostimulants with grid center X = 2.367, Y = -2.724, Z = 1.917) while a grid box measuring 20 Å × 20 Å × 20 Å was created for nanoparticles with grid center (X = 6.625,Y=5.556, Z= -4.253). In molecular docking investigations, the grid box is crucial for identifying the active or binding sites inside protein targets (Trott and Olson, 2009).

#### 2.6.3 Molecular Docking process

Auto-Dock Tools AutoDockTools-1.5.7 was used to dock salicylic acid (SA) and humic acid (HA) at the protein binding site (Trott and Olson, 2009). After ligand docking with the target was finished, the results were logged and arranged based on binding energy (ΔG) kcal/mol. Next, utilizing BIOVIA Discovery Studio 2021, the molecular docking interactions between docked compounds, such as salicylic acid (SA), humic acid (HA), and receptors (protein) were demonstrated (Murthy et al., 2019). However, in order to build the structure of a ZnO, we applied to the UFF force field and utilized Avogadro 1.2.0 to add hydrogen atoms, distribute partial charges, and reduce energy in order to minimize energy (El-Naggar et al., 2024). The docking was performed by the autodocking 4.2 program with 25 million evaluations, 500 population sizes, and 50 genetic algorithm runs. Chimera and Discovery Studio 2021 assessed the interactions between the ZnO NPs and amino acid residues after the docking procedure (Akram et al., 2021; El-Naggar et al., 2024).

### 2.7 Greenhouse experiment

#### 2.7.1 Time course of experiment

A semi-field experiment was conducted within the greenhouse of the Botany Department at Mansoura University to investigate the impact of various treatments on the growth and metabolism of tomato plants. Uniform tomato plantlets, 35 days old, were allocated into eight groups and transplanted into pots containing sterilized garden soil composed of clay and sand in a 2:1 volume ratio. Three replicates were used as three pots per treatment, each containing five tomato plants (n = 15 plants per treatment). Super phosphate was applied at a rate of 0.6 g per pot, equivalent to 357.14 kg per hectare, as recommended by the Ministry of Agriculture, Egypt. The treatments included: TO^−^ = negative control (Dist. water), TO^+^ = positive control (*F. oxysporum*), T1 = 0.5 mM SA, T2 = 0.6 mM SA, T3 = 100 mg/L HA, T4 = 150 mg/L HA, T5 = 250 mg/L ZnO-NPs, and T6 = 500 mg/L ZnO-NPs. Treatments were administered via foliar spray on the 40^th^ day. The inoculum was prepared by combining *F. oxysporum* cultures with a sterilized sand-cornmeal medium and subsequently added to pots at a ratio of 1g inoculum per 100g soil. Data collection of morphological, physiological and biochemical parameters of 70-day-old *S. lycopersicum* plants at the early fruiting stage were obtained under various treatments.

#### 2.7.2 Growth parameter estimation

Plant growth was evaluated by measuring the lengths of shoots and roots, as well as the fresh and dry weights of the plants. Furthermore, the number of branches, leaves, and fruits was documented. The fresh and dry weights were measured after washing and drying plant tissues at 80 °C for 48 hours.

#### 2.7.3 Biochemical and physiological parameters measurements

##### 2.7.3.1 Photosynthetic pigments

The photosynthetic pigments (chlorophyll *a*, chlorophyll b, and carotenoids) were estimated following the spectrophotometric method of Borisade et al (Borisade et al., 2017). Fresh leaf samples were homogenized in 10 mL of 80% acetone with MgCO_3_ to prevent acidification. Extractions were performed in the dark to minimize chlorophyll degradation. After centrifugation at 1788.8 × g for 5 minutes, the supernatant’s absorbance was measured at 480, 644, and 663 nm using a spectrophotometer against an 80% acetone blank. Pigment concentrations (μg/mg) were calculated using the equations:


Chlorophyll a=10.3×E663−0.918×E644

Chlorophyll b=19.7×E644−3.878×E663

Carotenoids=5.02×E480

Values were expressed as μg/g fresh weight.

##### 2.7.3.2 Estimation of total carbohydrates

Dried samples (0.1 g) were hydrolyzed in a boiling water bath with 5 mL of 2.5 N-HCl for 3 hours. After cooling, the solution was neutralized with solid sodium carbonate, diluted to 100 mL, and centrifuged. A 0.5 mL aliquot of the supernatant was diluted to 1 mL with distilled water, followed by the addition of 4 mL anthrone reagent. The mixture was heated for 8 minutes in a boiling water bath, rapidly cooled, and the absorbance was measured at 630 nm. Glucose standards were used to prepare a calibration curve (Sadasivam and Manickam, 1996).

##### 2.7.3.3 Determination of total protein

Fresh tomato leaves (0.2 g) were homogenized in 0.05M Tris HCL buffer (pH 8.0) using precooled mortars and pestles. After centrifugation, clear supernatants were either used immediately for protein assay or stored at -20 °C (Arulsekar et al., 1986). For each sample, 0.1 mL of supernatant and extraction buffer (blank) were prepared. To each tube, 3 mL of Bradford reagent was added, gently mixed, and the absorbance was measured at 595 nm after 5 minutes but within 1 hour. Protein concentrations (mg/g fresh weight) were calculated from a standard curve prepared using bovine albumin, accounting for dilution factors (Bradford, 1976).

##### 2.7.3.4 Estimation of total phenols

Total phenols were quantified using the Folin-Ciocalteu reagent as outlined by Malik and Singh (1980). Root samples (0.5–1.0 g) were homogenized in 80% ethanol (10× sample volume) and centrifuged at 10,000 g for 20 minutes. The supernatant was collected, and the residue was re-extracted five times. After evaporation to dryness, the residue was dissolved in 5 mL distilled water. A 0.2 mL aliquot was diluted to 3 mL with distilled water, followed by the addition of 0.5 mL Folin reagent. After 3 minutes, 2 mL of 20% Na_2_CO_3_ was added, mixed, and heated in boiling water for 1 minute. Absorbance was measured at 650 nm. A standard curve using varying Catechol concentrations was used for phenol quantification.

##### 2.7.3.5 Determination of antioxidant activity by DPPH radical

The antioxidant properties of tomato leaves were evaluated using the DPPH radical assay as described by Rosidah et al (Rosidah et al., 2008). Methanolic extracts (1 mL) were combined with 2 mL of a 0.1 mM DPPH solution, and this was done in triplicate. Control samples consisted of methanol and DPPH, while blanks contained only methanol. After a 30-minute incubation period in darkness, the absorbance was recorded at 517 nm. The percentage of DPPH radical inhibition was determined using the formula:


Inhibition (%)=[A Control−A Sample)/A Control]×100,

where A Control represents the absorbance of the DPPH solution with methanol, and A Sample is the absorbance of the DPPH solution with the leaf extract. This technique measures the antioxidant capacity of the samples.

##### 2.7.3.6 Total antioxidant capacity

Total antioxidant capacity was determined using the molybdate-reducing method (Prieto et al., 1999). Methanolic leaf extracts (0.3 mL) were mixed with 2.7 mL phosphomolybdenum reagent (4 mM ammonium molybdate, 0.6 M sulfuric acid, and 28 mM sodium phosphate) in capped test tubes. The mixture was incubated at 95°C for 90 min, cooled to room temperature, and absorbance measured at 695 nm using a UV-visible spectrophotometer against a methanol blank. Results were stated as mg ascorbic acid equivalent per g dry weight of leaves, based on a standard curve prepared with ascorbic acid.

### 2.8 Statistical analysis

In this study, morpho-physiological data were assessed using a randomized complete block design with three replicates. Tests for normality and homogeneity of variance were conducted, and qualitative variables are presented as absolute values and percentages. ANOVA was performed using SPSS (version 22.0, IBM Corp., Armonk, NY, USA), with treatments considered as independent variables, and the results were expressed as means ± standard error. A significance level of *P* ≤ 0.05 was established by *Post-hoc* comparisons using Tukey’s HSD test, and figures were plotted using SigmaPlot 14.0 (Systat Software, Inc., San Jose, CA, USA). Graphical representations of all the examined parameters were created using JMP version 16 (SAS Institute Inc., Cary, NC, USA). Correlation coefficient matrices were calculated to produce scatter plots and heatmaps for morpho-physiological characteristics. Principal component analysis (PCA biplot) was employed to evaluate the distribution of treatments based on quantitative data, with treatments grouped using clustergram analysis. Hierarchical co-clustering (dendrogram) and heatmaps derived from combined morpho-physiological data were generated using Ward’s method in JMP version 16.

## 3 Results

### 3.1 Morphological and physical characterizations of ZnO-NPs

ZnO-NPs dispersed in ultrapure water demonstrated an absorption peak at 300 nm, which is recognized to be the intrinsic Zn-O bandgap. Additionally, a broad absorption band extending to longer wavelengths was observed, likely due to electronic cloud movement (**Supplementary Figure S1A**). TEM analysis revealed irregular round, spherical, and hexagonal shapes with average sizes ranging from 17 to 50 nm, as depicted in **Figure S1B**. FTIR absorption peaks were identified within the ranges of 3750–3450 cm^-^¹, corresponding to hydroxyl group stretching, approximately 2922 cm^-^¹ for CH_2_ and CH_3_ stretching, 1400–1586 cm^-^¹ for C=O stretching, around 1000 cm^-^¹ for CH_2_ and CH_3_ bending, and 415–480 cm^-^¹ for Zn-O stretching. These observations underscore the presence of functional groups and the interatomic vibration characteristics of ZnO nanoparticles (**Supplementary Figure S1C**).

### 3.2 Microbiological analyses

#### 3.2.1 Molecular identification of *F. oxysporum*

**Figure 1:**
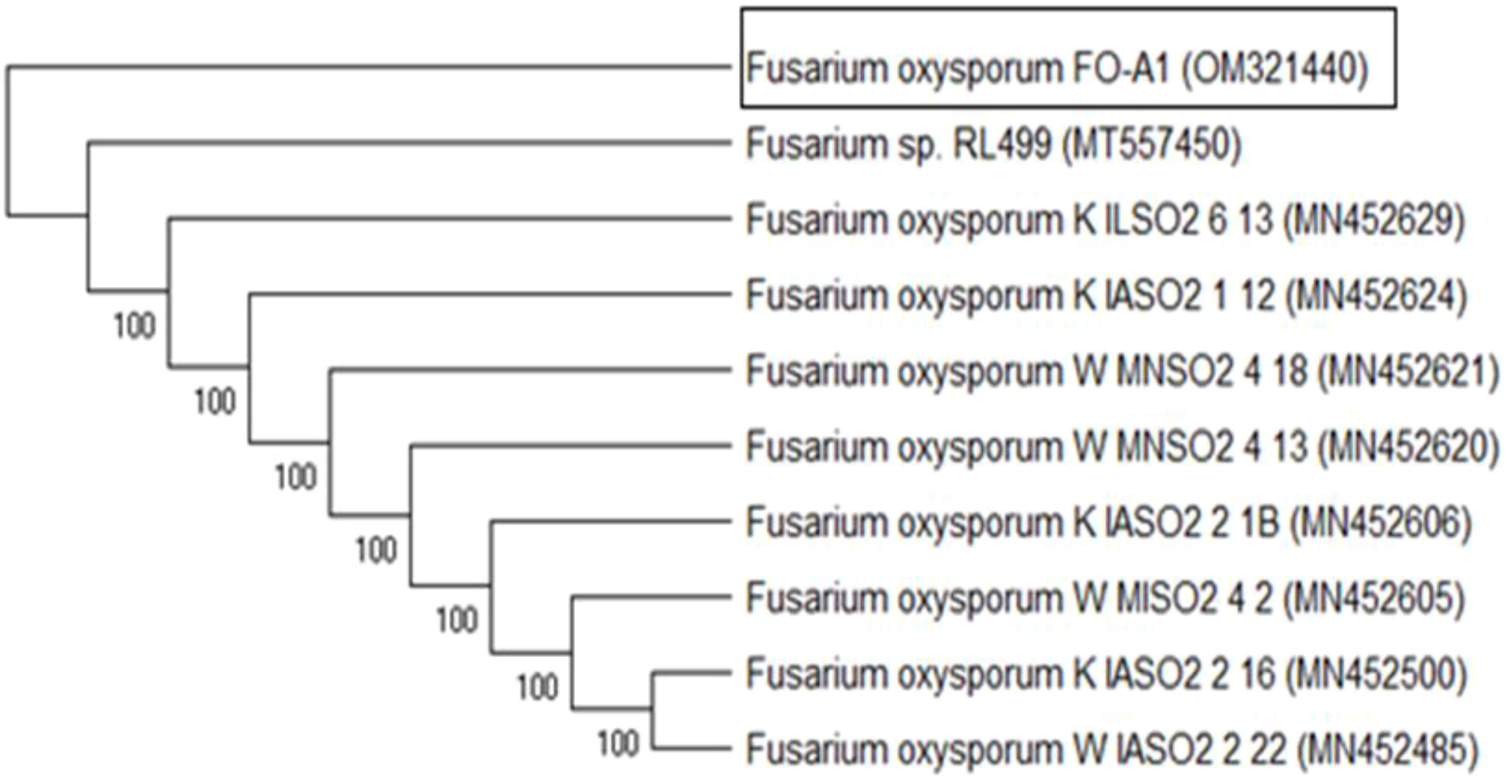
Molecular identification of *F. oxysporum*. Phylogenetic tree constructed using ITS region sequences to confirm species identity. The tree was generated using the Neighbor-Joining method with 1000 bootstrap replicates to assess branch reliability. GenBank accession numbers for reference sequences are shown in brackets. The isolate under study is highlighted, demonstrating its clustering within the *F. oxysporum* clade.

Genomic DNA was extracted from the fungal mycelia, and the Internal Transcribed Spacer (ITS) region was amplified, purified, and sequenced using specific primers. BLASTn analysis of the sequences against the GenBank database confirmed a 100% match with *F. oxysporum* ITS sequences. Phylogenetic analysis (**Figure 1**) categorized the isolates into two clusters: one containing FO-A1 (OM321440) and the other comprising nine species. Isolate RL499 (MT557450) demonstrated the highest similarity to the studied strain, thereby validating the molecular identification and corroborating the morphological findings.

#### 3.2.2 *In vitro* antifungal activity of SA, HA and ZnO-NPs

**Figure 2:**
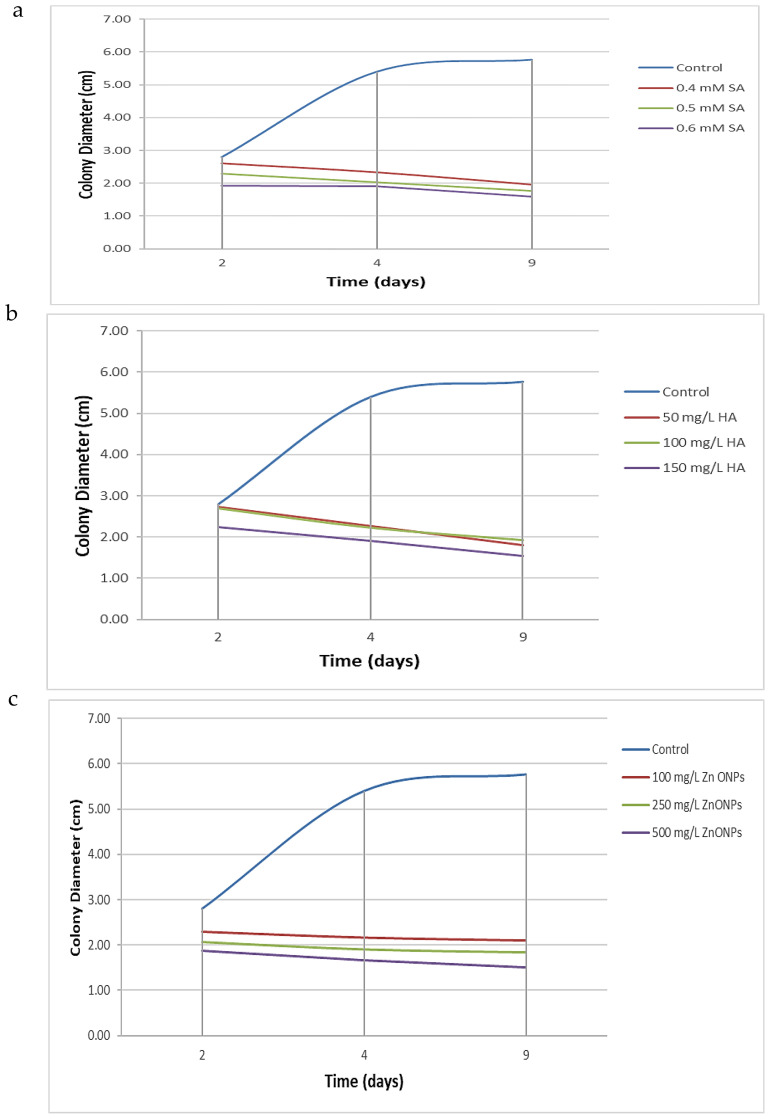
Kinetic of different treatment over time. **(a)** Effect of varying concentrations of SA, **(b)** HA, and **(c)** Effect of ZnO-NPs on the mycelial growth diameter of *F. oxysporum* after 2, 4, and 9 days of incubation. Each curve represents the mean growth response at specific time intervals, illustrating the inhibitory trends of each treatment compared to the untreated control.

**Figure 3:**
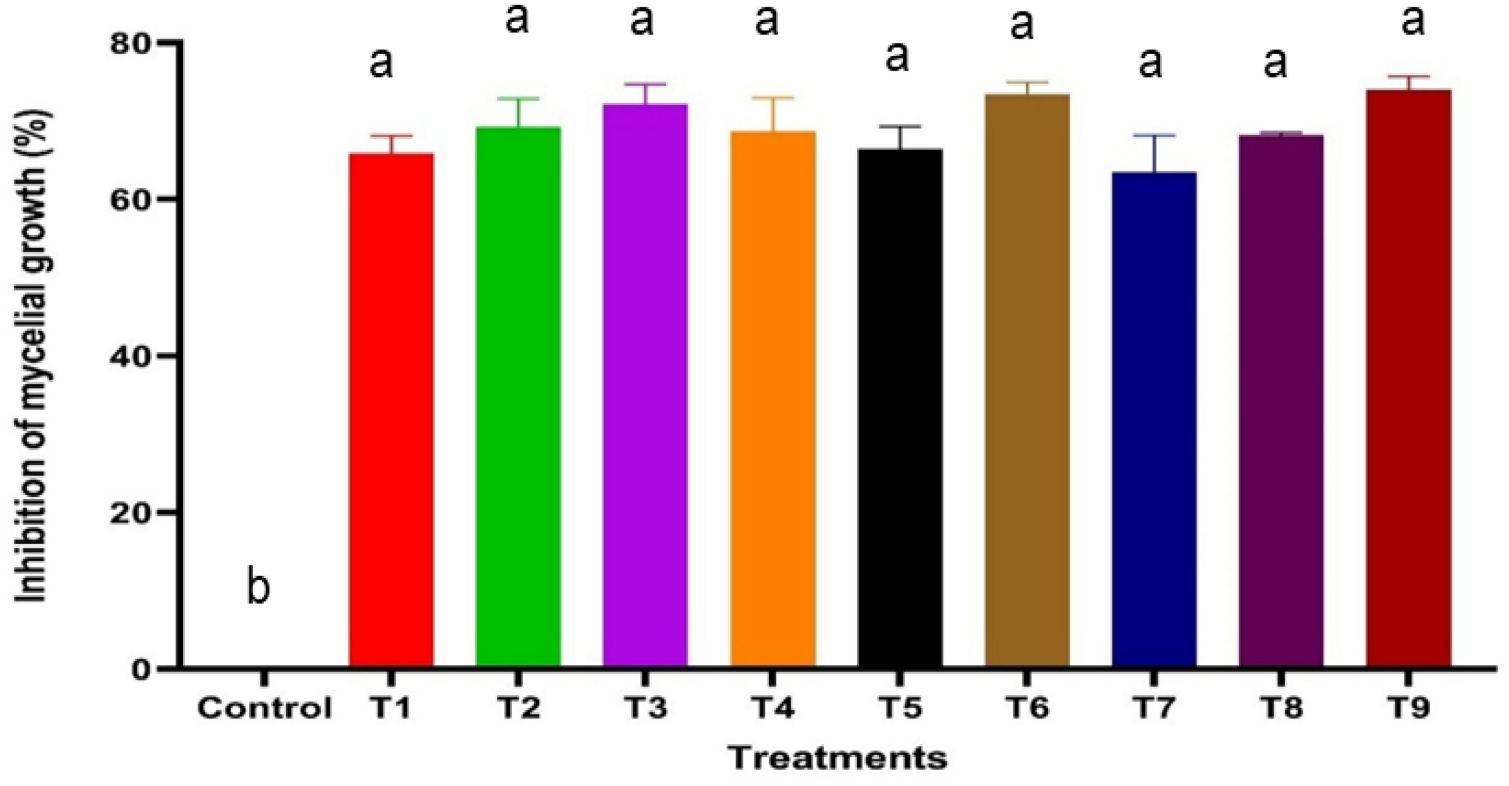
Effect of varying concentrations of SA, HA, and ZnO NPs on the inhibition of *F. oxysporum* mycelial growth after 9 days of incubation. Error bars represent standard deviations, and different superscript letters indicate statistically significant differences at *P* ≤ 0.05 based on ANOVA followed by *post-hoc* tests. Treatments with higher concentrations generally exhibited greater inhibition, highlighting the dose-dependent effect of SA, HA, and ZnO-NPs. T1, 0.4 mM SA; T2, 0.5 mM SA; T3, 0.6 mM SA; T4, 50 mg/L HA; T5, 100 mg/L HA; T6, 150 mg/L HA; T7, 100 mg/L ZnO NPs; T8, 250 mg/L ZnO NPs; T9, 500 mg/L ZnO NPs.

The antifungal efficacy of SA, HA, and ZnO-NPs was evaluated at various concentrations, and the results are detailed in **Table 1**, **Figures 2**, **3** and **Supplementary Figure S2**. Significant differences were observed in the inhibition of *F. oxysporum* mycelial growth. ZnO-NPs demonstrated the most substantial antifungal activity, achieving inhibition percentages of 73.99% at 500 mg/L and 68.21% at 250 mg/L. The inhibition percentages of SA at concentrations of 0.6 mM and 0.5 mM resulted in inhibition percentages of 72.25% and 69.36%, respectively, while 150 mg/L and 100 mg/L HA exhibited inhibition percentages of 73.41% and 66.47%, respectively. Lower concentrations of all treatments did not show significant differences compared with the control. *In vitro* antifungal assays were conducted on PDA at 25°C. The kinetics of different treatments were detected over time as SA inhibited mycelial growth in a concentration-dependent manner, reducing the control colony diameter from 5.7 cm to 1.7 cm at 0.6 mM (**Figure 2a**). HA also demonstrated dose-dependent effects, reducing colony diameter from 5.7 cm in the control to 1.53 cm at 150 mg/L (**Figure 2b**). Similarly, ZnO-NPs displayed a linear increase in inhibitory effects with higher concentrations, with 500 mg/L being the most effective (**Figure 2c**). These findings substantiate the efficacy of these treatments, particularly at elevated concentrations, in suppressing the growth of *F. oxysporum* (**Figure 3**).

**Table 1:** Effect of different concentrations of SA, HA, and ZnO-NPs on *F. oxysporum* mycelial growth diameter and antifungal activity inhibition percentages at 2, 4, and 9 days.

Treatments	Mycelial growth diameter of *F. oxysporum* (cm)			Inhibition of *F. oxysporum* mycelial growth after 9 days (%)
	After 2 days	After 4 days	After 9 days	
Control	2.80 ± 0.12^a^	5.40 ± 0.10^a^	5.77 ± 0.12^a^	0.00 ± 0.00^b^
T1	2.60 ± 0.12^a^	2.33 ± 0.09^b^	1.97 ± 0.09^b^	65.90 ± 1.28^a^
T2	2.30 ± 0.06^b^	2.03 ± 0.03^b^	1.77 ± 0.09^b^	69.36 ± 2.05^a^
T3	1.93 ± 0.07^b^	1.90 ± 0.06^b^	1.60 ± 0.06^b^	72.25 ± 1.45^a^
T4	2.73 ± 0.09^a^	2.27 ± 0.12^b^	1.80 ± 0.12^b^	68.79 ± 2.47^a^
T5	2.70 ± 0.06^a^	2.23 ± 0.07^b^	1.93 ± 0.09^b^	66.47 ± 1.65^a^
T6	2.23 ± 0.09^b^	1.90 ± 0.06^b^	1.53 ± 0.03^b^	73.41 ± 0.89^a^
T7	2.30 ± 0.15^b^	2.17 ± 0.12^b^	2.10 ± 0.12^b^	63.58 ± 2.72^a^
T8	2.07 ± 0.03^b^	1.90 ± 0.06^b^	1.83 ± 0.03^b^	68.21 ± 0.18^a^
T9	1.87 ± 0.03^b^	1.67 ± 0.07^b^	1.50 ± 0.06^b^	73.99 ± 1.02^a^
LSD (5%)	**0.2564**	**0.2474**	**0.2625**	**4.9817**

The results represent the mean of triplicates ± standard error (SE). LSD stands for Least Significant Difference. One-way ANOVA was used, and different superscript letters indicate significant differences based on [Tukey’s HSD test] at P ≤ 0.05. Treatment codes are as follows: T1, 0.4 mM SA; T2, 0.5 mM SA; T3, 0.6 mM SA; T4, 50 mg/L HA; T5, 100 mg/L HA; T6, 150 mg/L HA; T7, 100 mg/L ZnO-NPs; T8, 250 mg/L ZnO-NPs; and T9, 500 mg/L ZnO-NPs.

#### 3.2.3 *In vivo* inhibition of *F. oxysporum*

All treatments significantly reduced disease incidence and severity in plants infected with *F. oxysporum*. The most effective treatment was T6 (500 mg/L ZnO-NPs), which reduced disease incidence to 65% and disease severity to 1.0, compared to the inoculated control (TO^+^), where disease incidence and severity were 100% and 2.0, respectively (**Table 2**). While the untreated control (TO^-^) showed no significant changes, disease incidence and severity increased in plants infested with the fungus. However, the application of various treatments led to a marked reduction in both parameters, highlighting their efficacy in mitigating the effects of *F. oxysporum.*

**Table 2:** Disease incidence and severity indices of *F. oxysporum*-infested plants after different treatments.

Treatments	Percent disease incidence (PDI)	Disease severity index (DSI)
TO^-^	0.0 ± 0.0	0.0 ± 0.0
TO^+^	100.0 ± 0.0	2.0 ± 0.7
T1	85.0 ± 13.7	1.0 ± 0.0
T2	83.0 ± 13.7	1.0 ± 0.0
T3	87.0 ± 0.0	1.0 ± 0.0
T4	75.0 ± 0.0	1.0 ± 0.0
T5	78.0 ± 11.2	1.0 ± 0.0
T6	65.0 ± 13.7	1.0 ± 0.0

Results are presented as mean of triplicates ± standard error (SE). Treatments: TO^−^, negative control; TO^+^, positive control; T1, 0.5 mM SA; T2, 0.6 mM SA; T3, 100 mg/L HA; T4, 150 mg/L HA; T5, 250 mg/L ZnO-NPs; and T6, 500 mg/L ZnO-NPs.

### 3.3 *In silico* molecular docking studies

#### 3.3.1 Biostimulants interactions

**Figure 4:**
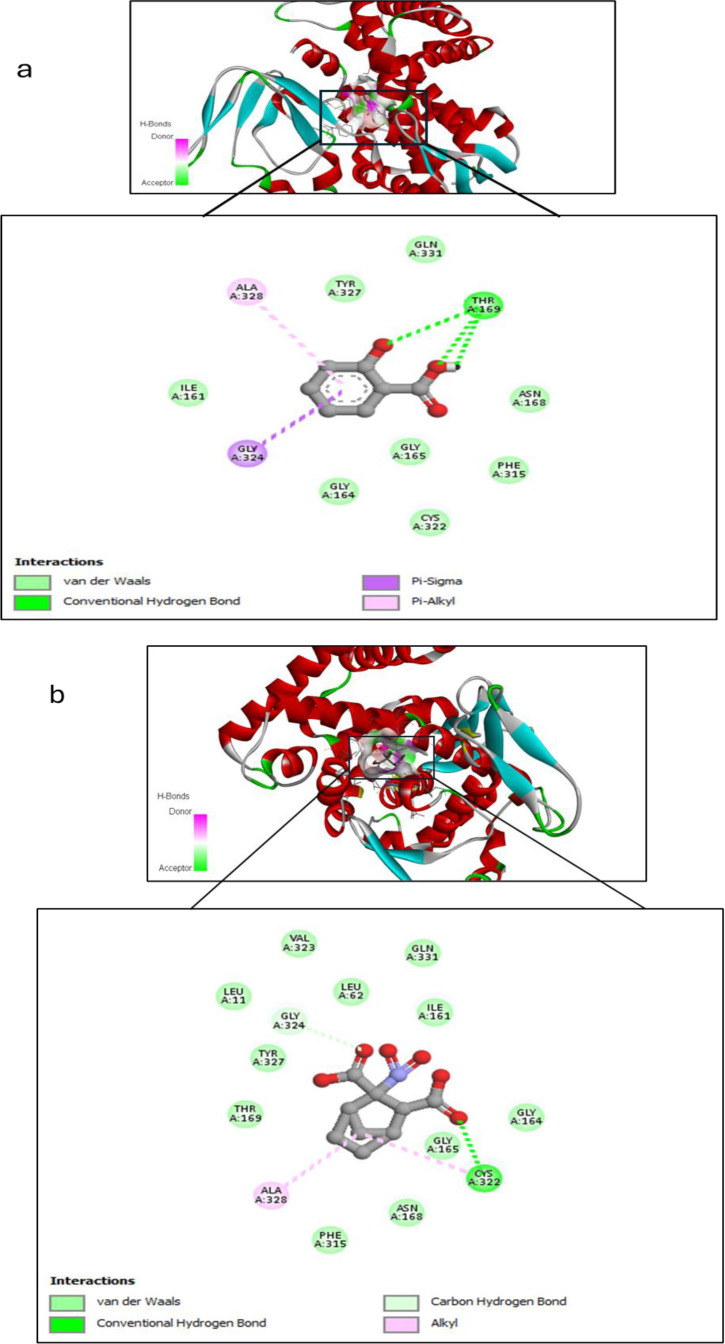
Molecular docking interaction between **(a)** Salicylic acid and receptor; and **(b)** Humic acid and receptor, with best mod RMSD = 0.0.

Salicylic acid and humic acid both interact with sterol 14 alpha-demethylase (CYP51), a key enzyme in fungal ergosterol biosynthesis (**Table 3**). Salicylic acid exhibits a binding affinity of –5.6 kcal/mol to sterol 14 alpha-demethylase (**Figure 4a**) indicating high and stable interaction with the enzyme’s active site, while Humic acid shows lower binding affinity of –2.4 kcal/mol for the same protein (**Figure 4b****).** The difference in binding affinity implies that salicylic acid has a better fit and potentially stronger inhibitory action against sterol 14 alpha-demethylase than humic acid. Both compounds primarily interact with amino acid residues in the enzyme’s active site through hydrophobic contacts and hydrogen bonding. However, the docking results indicate that salicylic acid exhibits a comparatively stronger binding affinity, suggesting greater antifungal potential against Fusarium via CYP51 inhibition. In contrast, Voriconazole—a triazole-based antifungal agent—functions as a cytochrome P450 inhibitor and is widely used to treat severe fungal infections caused by Fusarium spp.

**Table 3:** The binding affinity of biostimulants with protein receptors.

Molecules	Binding energy (Kcal/mol)	Estimated inhibition constant, Ki (mM)	Metal chelating residues
CYP51	-2.19	24.97	ZnO-ARG A:321 ZnO-LYS A4

#### 3.3.2 Zinc oxide nanoparticles Interaction

**Figure 5:**
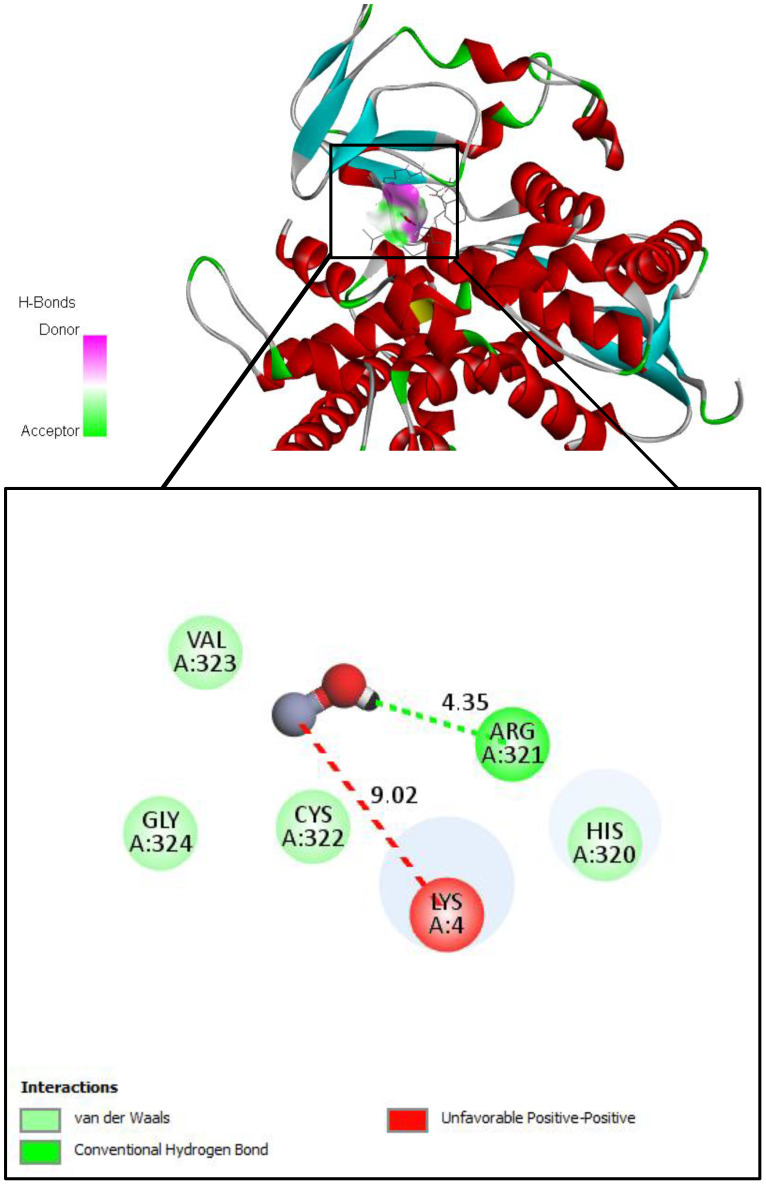
Molecular docking and interactions between ZnO nanoparticles and the amino acid residues of CYP51 protein. With the grid center coordinates (6.625 -5.556,-4.253) and the number of requested GA dockings = 50 runs with distance 4.35 Å twice, with ARG and 9.02 Å twice with LYS.

On the other hand, we used Auto-dock parameter version 4.2 in the molecular docking process to investigate the preliminary interactions between the ZnO- NPs and protein targets, such as CYP51 protein. **Table 4** depicts the binding of ZnO- NPs with the protein during the docking approach. During the docking process, the binding energy was less than 0 kcal/mol, indicating that it was more likely to interact with the binding residues of the target proteins at its free energy as shown in **Table 2**. During the docking process, number of conformations = 50, RMSD cluster analysis will be performed using the ligand atoms only (3/3 total atoms). Outputting structurally similar clusters, ranked in order of increasing energy. Number of distinct conformational clusters found = 6, out of 50 runs, Using an RMSD-tolerance of 2.0 Å. The interaction analysis showed that ZnO-NPs can interact with the ARG amino acids, through the conventional hydrogen bond as shown in **Figure 5**.

**Table 4:** Molecular docking analysis of ZnO nanoparticles.

Biostimulants	Target protein	Binding affinity Kcal/mol	Amino acids
Salicylic acid	sterol 14 alpha-demethylase	-5.0	HIS A:377, LEU A:376, SER A:378, MET A:508
Humic acid		-2.4	TYR A:132, LEU A:376, TYR A:118
Voriconazole (Antifungal Reference		-1.2	CYS A:470, LEU A:376, LEU A:121, PHE A:380, HIS A:377, SER A:388, MET A:508

### 3.4 Physiological analyses

#### 3.4.1 Impact of treatments on morphological traits of *S. lycopersicum*

**Figure 6:**
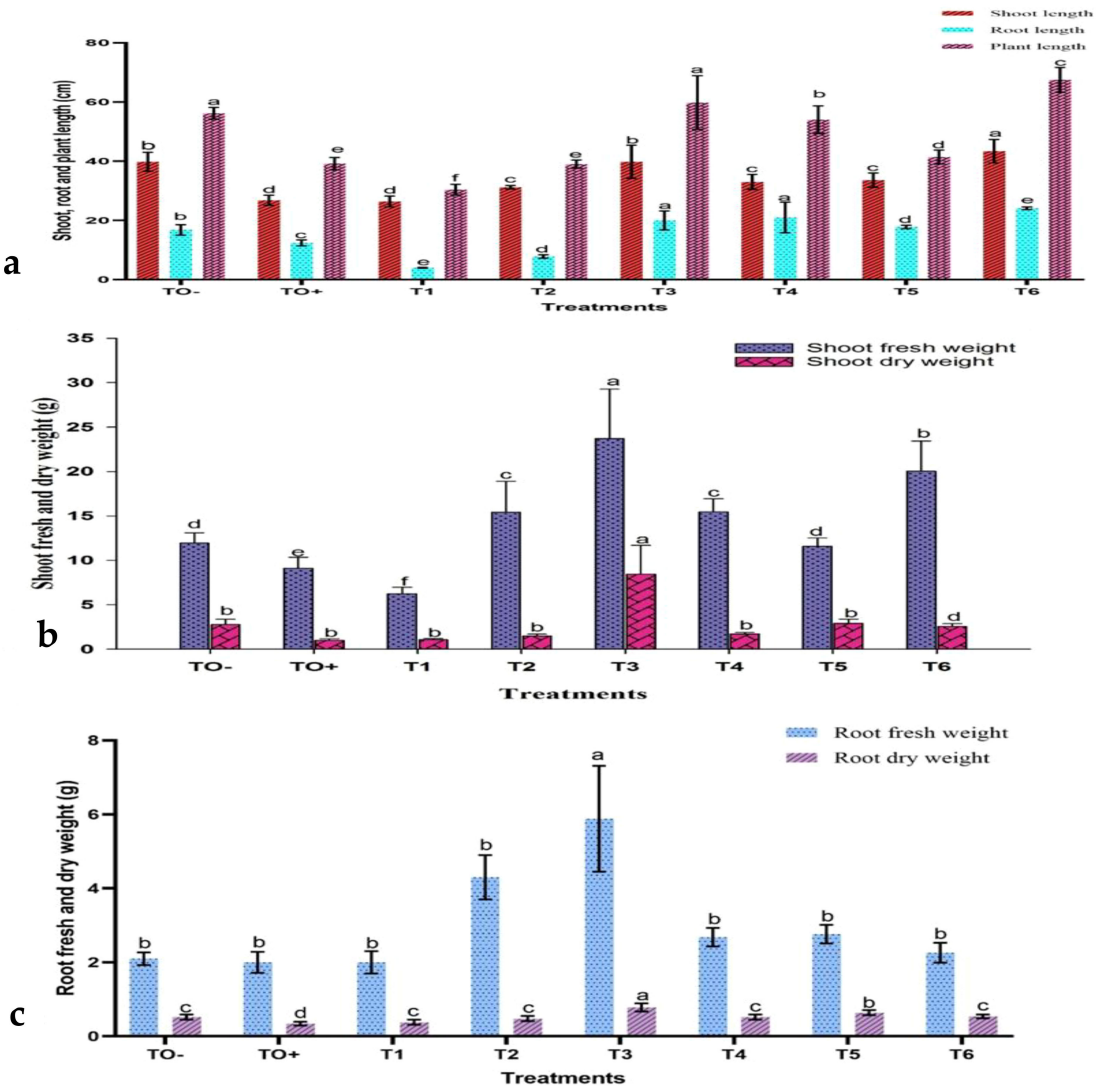
The effect of treatments on **(a)** shoot, root, and plant height; **(b)** shoot fresh and dry weights; and **(c)** root fresh and dry weights of 70-day-old *S. lycopersicum* plants. Error bars with different superscript letters indicate significant differences at *P* ≤ 0.05.

The data obtained from the greenhouse experiment indicated significant modifications in the morphological characteristics of 70-day-old *S. lycopersicum* plants at the early fruiting stage under various treatments (**Supplementary Figure S3**). Growth parameters, including shoot and root lengths, leaf count, fresh and dry weights, and number of fruits and branches, were measured. Plants infected with *F. oxysporum* exhibited stunted growth, as evidenced by shorter root lengths, reduced plant height, smaller leaf area, decreased fresh and dry weights, and leaf chlorosis. Infected plants demonstrated significant reductions in both shoot and root lengths (**Figure 6a**) as well as in fresh and dry weights (**Figures 6b, c**).

Nevertheless, various treatments resulted in enhanced growth characteristics, to varying degrees. The application of 500 mg/L ZnO-NPs (T6) yielded the greatest shoot length (43.40 cm), tallest plant height (67.50 cm), and highest leaf count (56.40 cm). HA at concentrations of 150 mg/L (T4) and 100 mg/L (T3) produced the longest root lengths (21.00 cm and 20.00 cm, respectively). In the same manner, 100 mg/L HA treatment (T3) resulted in the highest shoot fresh and dry weights (23.72 g and 8.44 g) and root fresh and dry weights (5.88 g and 0.78 g).

**Figure 7:**
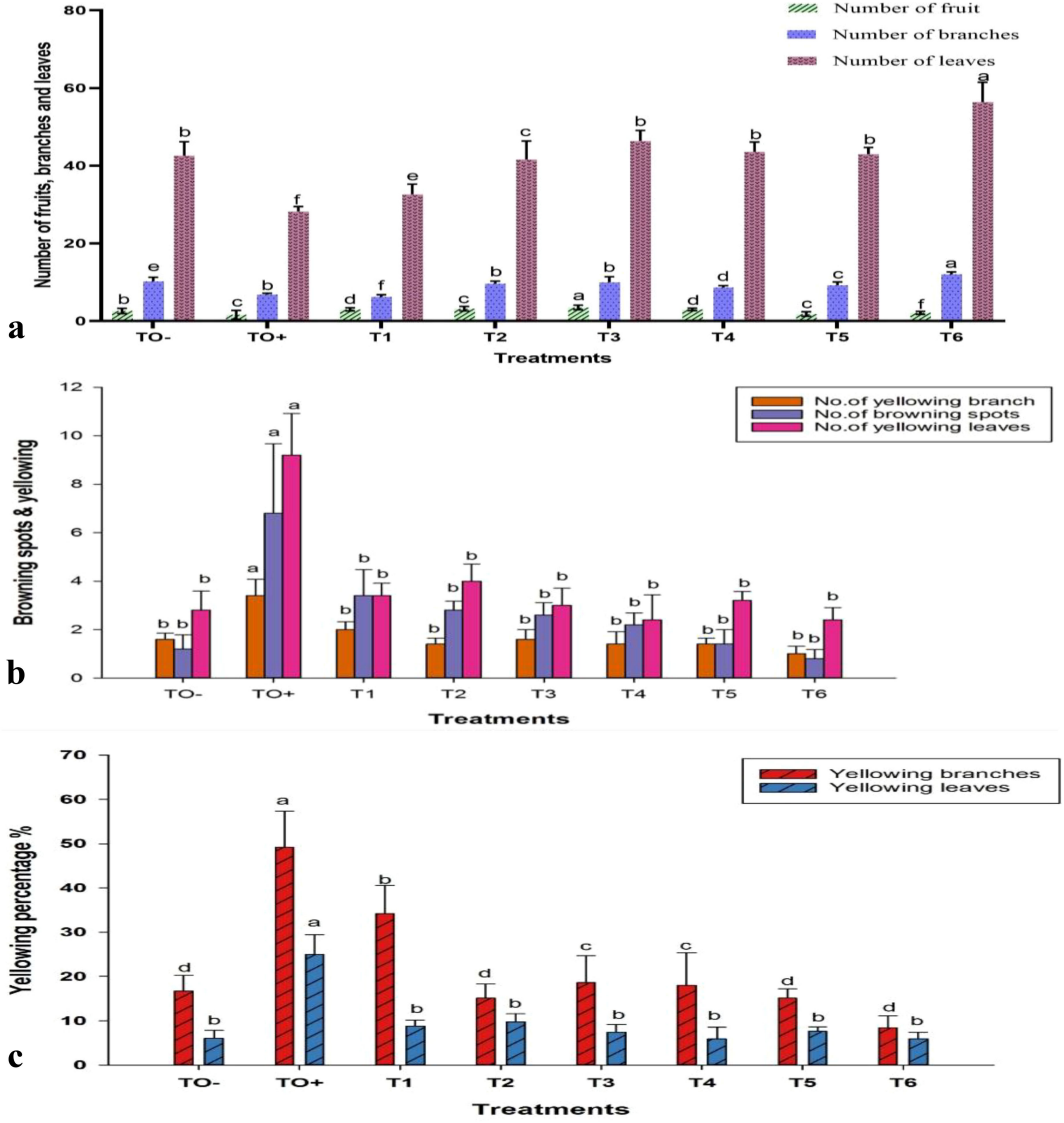
Effect of different treatments on **(a)** number of fruits, branches, and leaves; **(b)** browning spots and yellowing branches and leaves; and **(c)** percentage of yellowing branches and leaves in 70-day-old *S. lycopersicum*. Error bars with different superscript letters indicate significant differences at *P* ≤ 0.05.

In terms of branching and fruit production, the treatment T6 demonstrated a significant increase in the number of branches, reaching a total of 12.00. In contrast, treatments T1, T2, and T4 notably enhanced fruit production, with T2 (0.6 mM SA) achieving the highest fruit yield of 3.20. Conversely, the untreated control (TO^+^) infected with *F. oxysporum* exhibited reduced growth and fruiting, whereas the negative control (TO^−^) had the highest number of leaves (46.80). These treatments significantly bolstered plant defense against *F. oxysporum*, resulting in improved growth and productivity (**Figure 7a**). Exposure to *F. oxysporum* (TO^+^) resulted in a significant increase in browning spots and yellow, number of branches and leaves, compared to the negative control (TO^−^) and treated plants. TO^+^ plants demonstrated the highest degree of yellowing, with 3.40 branches, 9.20 leaves, and 49.17% and 24.98%, respectively. Among the treatments, 500 mg/L ZnO-NPs (T6) exhibited the least impact, with only 1.00 browning spots, 2.4 yellowed branches, and 0.80 yellowed leaves recorded (**Figures 7b, c**).

**Figure 8:**
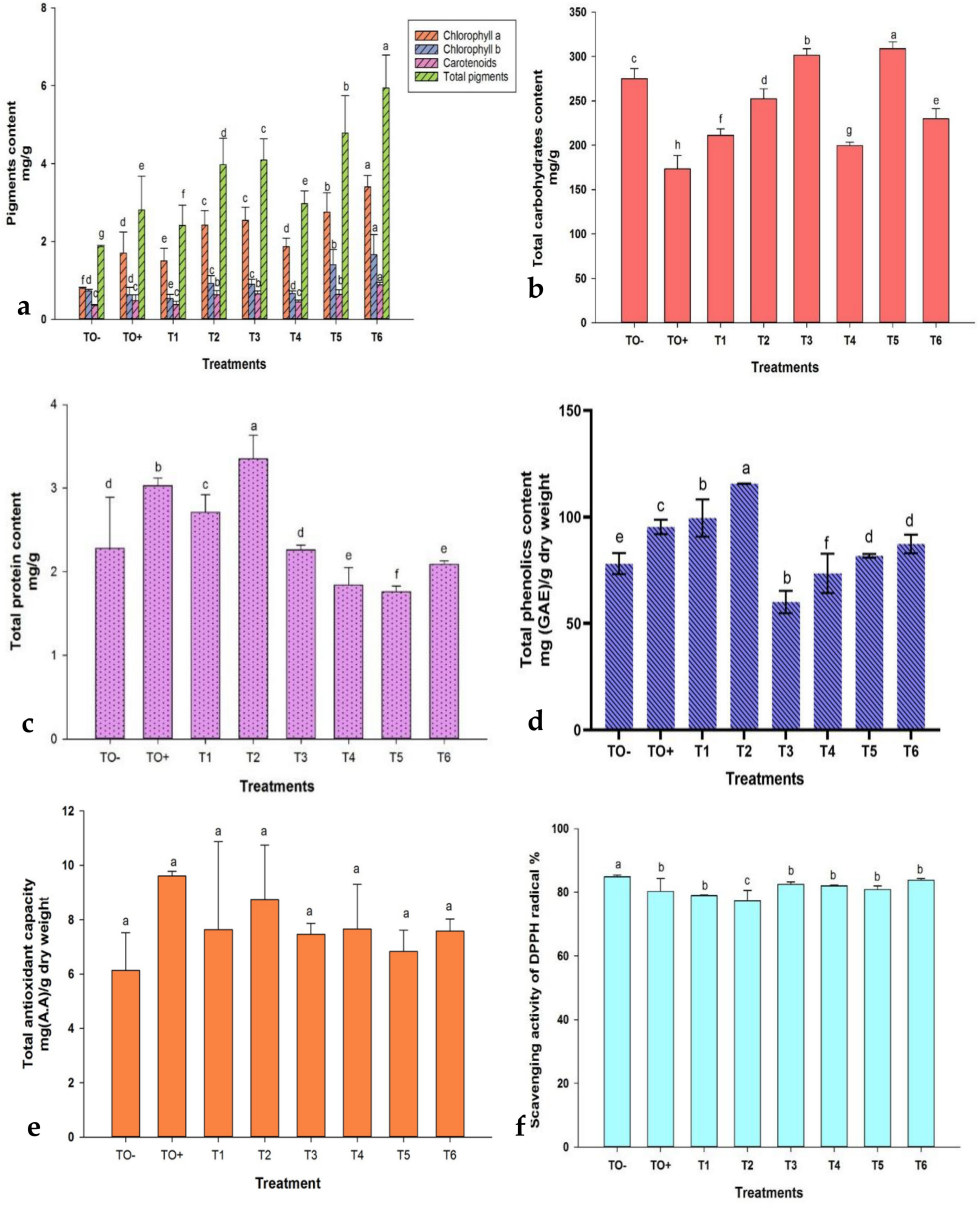
Effect of different treatments on **(a)** Photosynthetic pigments (chlorophyll *a*, chlorophyll *b*, carotenoids, and total pigments); **(b)** total carbohydrates; **(c)** total protein; **(d)** total phenolic content; **(e)** total antioxidant capacity; and **(f)** DPPH scavenging activity in 70-day-old *S. lycopersicum*. Error bars with different superscript letters denote significant differences at *P* ≤ 0.05.

Chlorophyll *a* exhibited a significant increase across all treatments, whereas chlorophyll *b* demonstrated a notable decrease in T1, non-significant decrease in TO^+^ and T4, and significant increase in the remaining treatments. The administration of 500 mg/L ZnO-NPs (T6) resulted in the highest pigment concentrations, with chlorophyll *a*, chlorophyll *b*, carotenoids, and total pigments measuring 3.40, 1.66, 0.88, and 5.94 mg/g, respectively. In contrast, the lowest concentrations of chlorophyll *a*, carotenoids, and total pigments were observed in TO^−^, with values of 0.79, 0.35, and 1.87 mg/g, respectively, while T1 (0.5 mM SA) exhibited the lowest chlorophyll b at 0.53 mg/g (**Figure 8a**).

The total carbohydrate content significantly increased in T3, T5, and T6, with T5 achieving the highest level at 308.97 mg/g (**Figure 8b**). Protein content varied, with significant increases in TO^+^, T1, and T2, where T2 (0.6 mM SA) recorded the highest value at 3.35 mg/g, whereas T5 had the lowest protein content at 1.76 mg/g (**Figure 8c**). The total phenolic content (TPC) was significantly affected by the treatments, decreasing in T3 and T4 but increasing in the others. T2 (0.6 mM SA) registered the highest TPC (115.63 mg/g), while T3 recorded the lowest (60.10 mg/g) (**Figure 8d**). Antioxidant capacity was highest in TO^+^ (9.61 mg/g) and lowest in TO^−^ (6.14 mg/g) (**Figure 8e**). DPPH scavenging activity showed a reverse trend, with T2 exhibiting the highest antioxidant activity (77.35%) and TO^−^ the lowest (84.87%) (**Figure 8f**).

#### 3.4.4 Combined correlation of morpho-physiological and biochemical data

**Figure 9:**
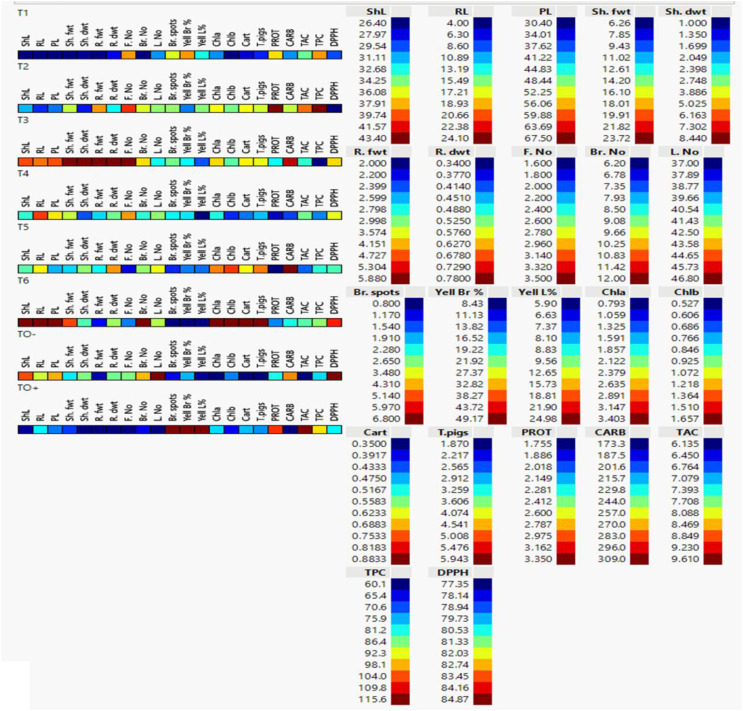
Cell plot of 22 morpho-physiological and biochemical traits of 70 days-old *S. lycopersicum* plants under eight different treatments. Data are presented as means of three replicates; low values are represented by dark blue and its gradients, while high values are represented by dark red (legend on the right). ShL, shoot length; RL, root length; PL, plant length; Sh. fwt, shoot fresh weight; Sh. dwt, shoot dry weight; R. fwt, root fresh weight; R. dwt, root dry weight; F. No, fruit number; Br. No, branch number; L. No, leaf number; Br. Spots, browning spots; Yell Br%, branch yellowing percentage; Yell L%, leaf yellowing percentage; Chl *a*, chlorophyll *a*; Chl *b*, chlorophyll *b*; Cart, carotenoids; T. pigs, total pigments; PROT, protein; CARB, total carbohydrate content; TAC, total antioxidant content; TPC, total phenolic content; and DPPH, 2,2-diphenyl-1-picrylhydrazyl.

Analysis of cell plots for 22 characteristics treated *S. lycopersicum* plants revealed significant differences among treatments. TO^+^ (Inoculated control), which marked in dark blue, showed the lowest levels of ShL, Sh. dwt, R. fwt, R. dwt, F. No, Br. No, L. No, Chl *b* and CARB. In the contrast, TO^+^ showed the highest values of Br. Spots, Yell Br, Yell L% and TAC which marked with dark red and the values of PROT, TPC, DPPH, Chl *a*, Cart and T. pigs was found as moderate colors between red to blue. TO^-^ (negative control) showed the contrary value of the tested parameters of TO +. TO^-^ showed the lowest values of Br. Spots, Yell Br, Yell L%, Chl a, Chl *b*, Cart, T. pigs and TAC which marked with dark blue, while the highest values L. No and DPPH which marked with dark red as illustrated (**Figure 9**).

#### 3.4.5 Multivariate analyses

##### 3.4.5.1 Heatmap and scatter plot correlation

**Figure 10:**
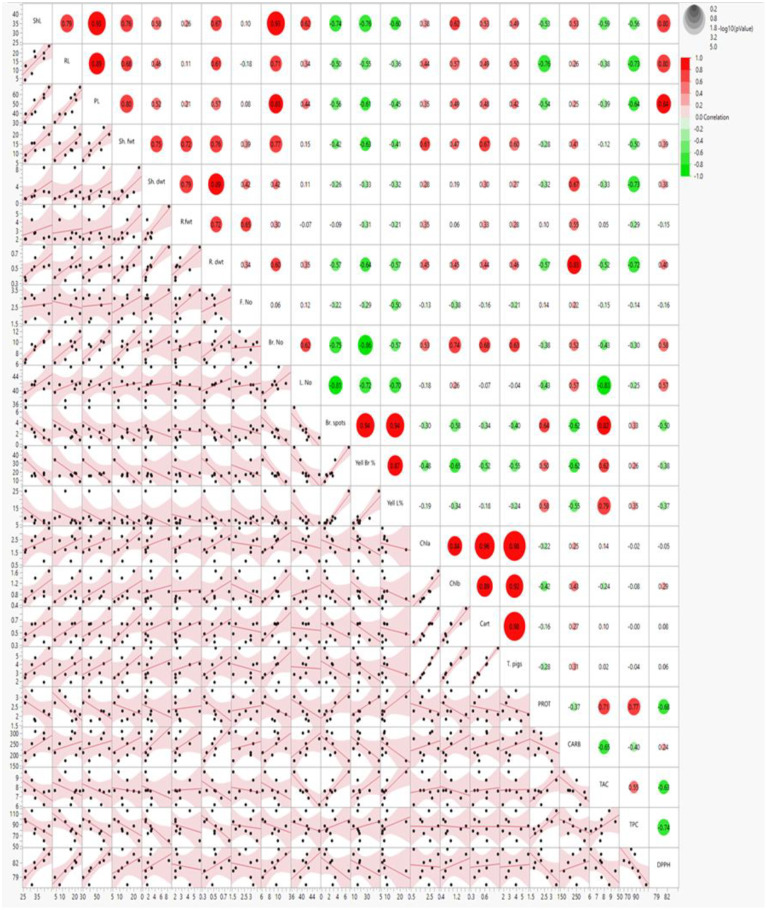
Scatter plot and heatmap correlation matrix of morpho-physiological and biochemical parameters in 70-day-old *S. lycopersicum* plants under eight treatments. Correlation levels are color-coded: red for high correlation, green for low (see scale in upper-right corner). Abbreviations as defined earlier.

Heatmap and scatter plot matrices have revealed significant associations between carbohydrates (CARB), pigments (chlorophyll *a*, chlorophyll b, carotenoids), DPPH and morphological attributes such as shoot and root lengths, fresh and dry weights, and the number of branches and leaves. The data revealed that CARB, pigmentation parameters (Chl *a*, Chl *b*, Cart and T. Pigs), and DPPH were strongly associated with each other, with all the morphological parameters (ShL, RL, PL, Sh. fwt, Sh. dwt, R. fwt, R. dwt, Br. No, L. No) except F. No, browning and yellowing symptoms (Br. Spots, Yell Br, Yell L%). In contrast, PROT, TAC and TPC were positively correlated to each other and with the browning spots and yellowing symptoms (Yell Br, Yell L%), whereas they were negatively correlated with all morphological parameters except F. No and weakly negative correlation with pigmentation (Chl *a*, Chl *b*, Cart and T. Pigs) as illustrated (**Figure 10**).

##### 3.4.5.2 Principal component analysis biplot

**Figure 11:**
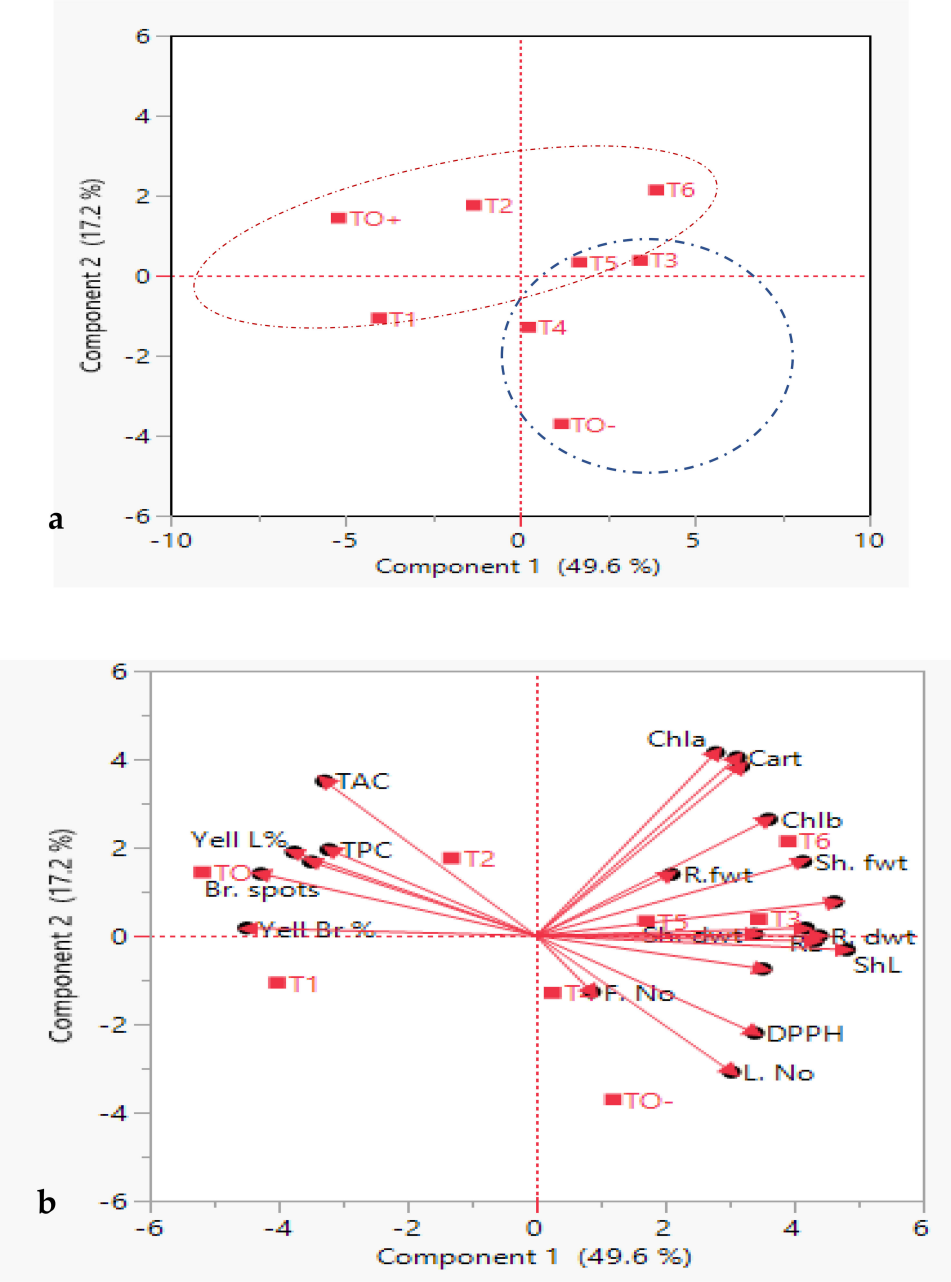
Principal component analysis (PCA), **(a)** eigenvalues and scatter plots, **(b)** biplot of treated *S. lycopersicum* plants on PC1 and PC2 based on morpho-physiological and biochemical traits. Blue and red dashed rings indicate two clusters. Dots represent treatments, and vectors (red arrows) represent parameters. Abbreviations are in earlier figures.

Principal Component Analysis (PCA) biplot analysis was employed to identify the primary factors contributing to the overall variation among the treatments. PCA effectively categorized the eight treatments into two distinct clusters: cluster I (blue group) included 4 treated *S. lycopersicum* plants (TO^-^, T3, T4 and T5), cluster II (Red group) had 3 treated plants (TO^+^, T1 and T2 andT6) which were distinct and well supported (**Figure 11a**).

**Figure 11b** depicts how the two first components, vector length and cosine of angle, were used to discriminate between treated *S. lycopersicum* plants. Notably, Chl *a*, Cart and T. pigs were the longest vectors, and the small angle between these vectors proved a significant strong positive correlation between these traits. Chla, Cart, T. pigs, Chl *b* and Sh. fwt. were the most effective parameters in separation between T5 and T6 *S. lycopersicum*. Moreover, vectors of ShL and R. dwt. showed the strong discrimination power for the evaluation between T3 and T5 *S. lycopersicum*. Also, vectors of DPPH and L. No. showed an effective power in differentiating between TO- and T4 *S. lycopersicum.* On the contrary, TAC and TPC traits had significant positive associations with one another but a negative correlation with L. No. and F. No. Sh. fwt. established a 90-degree angle with L. No., and a similar pattern was observed between Sh. fwt. and TAC, indicating that they were unlikely to be associated.

## 4 Discussion

*Fusarium oxysporum*, the 5^th^ most significant fungal plant pathogen, is the primary causative agent of Fusarium wilt in various crops, including tomatoes (Dean et al., 2012). Infected plants exhibit symptoms such as stunted growth, chlorosis, leaf necrosis, and wilting. These symptoms arise primarily due to pathogen infiltration through the root epidermis, followed by colonization of xylem vessels, resulting in water stress and vascular blockages (Singh et al., 2017). The persistence of the pathogen in the soil is facilitated by dormant chlamydospores (Khan et al., 2017), which complicates its management. Conventional control strategies include the burning of crop residues, the use of resistant plant varieties, soil fumigation, and the application of eco-friendly fungicides, including ZnO-NPs, SA, and HA (Karthika et al., 2020). Moreover, molecular analysis identified *F. oxysporum* as the fungal pathogen, with ITS gene sequences perfectly matching existing sequences (Adhikari et al., 2020).

Nanotechnology is becoming an important asset in agriculture, with uses including nano-fertilizers, pest control, and monitoring plant health (González-Merino et al., 2021). ZnO-NPs are noted for their strong germicidal effects, which are due to their high reactivity and small size (Elmer and White, 2018). In this study, TEM imaging showed ZnO-NPs in both spherical and rod shapes, consistent with previous research (González-Merino et al., 2021). FTIR analysis also verified the presence of functional biomolecules linked to these nanoparticles, such as Zn-O stretching vibrations in the 415–480 cm^-^¹ range, aligning with earlier studies (Muhammad et al., 2019; Jan et al., 2020),.

The antifungal efficacy of ZnO-NPs, SA, and HA was evaluated against *F. oxysporum*, with the inhibitory effects increasing at higher concentrations. ZnO-NPs at 500 mg/L, SA at 0.6 mM, and HA at 150 mg/L showed the highest inhibition percentages. These findings align with those of earlier studies that demonstrated the effectiveness of ZnO-NPs in controlling phytopathogens and enhancing crop protection (Dimkpa et al., 2013; González-Merino et al., 2021),. The nanoscale properties of ZnO-NPs not only improve their antimicrobial activity but also contribute to sustainable agricultural practices by promoting crop growth and yield (Kriti et al., 2020; Abdelaziz et al., 2022),.

Systemic acquired resistance (SAR) is a well-documented plant defense mechanism that depends on the accumulation of salicylic acid (SA) for signal initiation (Mandal et al., 2009). Effective management of tomato FW has been achieved through foliar applications of compounds such as validoxylamine A, which initiate SAR by elevating SA levels (Ishikawa et al., 2005). Exogenous application of SA enhances plant defenses by modulating gene expression, fortifying cell walls, and inducing oxidative bursts (Oostendorp et al., 2001). SA activates defense responses, including the SAR and immune pathways (Ding et al., 2018). Studies have demonstrated that SA treatment reduces plant mortality and leaf necrosis in *Arabidopsis* prior to exposure to *F. oxysporum* (Edgar et al., 2006), while also inhibiting fungal spore germination and hyphal development (Wu et al., 2008). Mandal, Mallick (Mandal et al., 2009) further demonstrated that SA applied as foliar sprays and root feeding can enhance resistance to *F. oxysporum* in tomatoes. Recent research indicates a synergistic effect of SA and rapamycin in preventing fungal invasion, with genetic modifications in transgenic potatoes effectively controlling wilt (Li et al., 2022).

Humic acid (HA), which is commonly utilized as a soil enhancer and plant growth promoter, has also demonstrated efficacy in disease control. HA supports plant growth by activating soil microorganisms, enhancing nutrient absorption, and promoting cell division (Abdel-Monaim et al., 2012). Additionally, its functional properties, such as the COOH group content, contribute to antifungal effects by inhibiting spore germination and fungal growth. Studies have confirmed the ability of HA to significantly reduce *F. oxysporum* infection and inhibit spore germination (Loffredo et al., 2007; Abdel-Monaim et al., 2011; Abdel-Monaim et al., 2012). These findings highlight the potential of HA to enhance natural resistance to plant pathogens. This study demonstrated that the treatments significantly reduced both the incidence and severity of wilt disease. The application of 500 mg/L ZnO-NPs was identified as the most effective, decreasing disease incidence to 65% and severity to 1, compared to the inoculated control, which exhibited 100% incidence and a severity of 2.0.

The integration of molecular docking in this study provided valuable insights into the potential mechanisms by which ZnO-NPs, SA, and HA exert antifungal effects against *F. oxysporum*. Docking analysis revealed preliminary interaction and binding affinities between these compounds and key pathogenic proteins involved in fungal virulence and survival, such as cell wall-degrading enzymes and signaling proteins. It has been demonstrated that several CYP51 paralogues, including CYP51A, CYP51B, and CYP51C, are expressed by Fusarium species, including *F. graminearum*. According to the study conducted by Fan, Urban (Fan et al., 2013) demonstrated that CYP51A and CYP51B paralogues predominantly facilitate sterol 14-demethylation and influence azole sensitivity and fungal growth, but CYP51C may be implicated in virulence without directly participating in sterol demethylation.

Moreover, the binding energy of biostimulants such as salicylic and humic acids with receptors suggests their potential as promising compounds for innovative antifungal drugs or as synergists to enhance the effectiveness of current fungicides. SA exhibited a binding affinity of –5.0 kcal/mol with CYP51, indicating a stable and high-affinity interaction with the enzyme’s active site. In comparison, HA showed a lower binding affinity of –2.4 kcal/mol, suggesting a comparatively weaker interaction. These differences imply that SA may exert a more potent inhibitory effect on CYP51 than HA. Both compounds primarily formed hydrophobic contacts and hydrogen bonds with key amino acid residues, supporting their potential as antifungal agents targeting ergosterol biosynthesis (Vermeulen et al., 2022). Focusing on different paralogues and conserved residues in CYP51 may be essential for sustainable management, given the global increase in Fusarium species’ resistance to traditional azoles (Naqvi et al., 2025).

ZnO-NPs demonstrated notable interactions with active sites of fungal proteins and may have the potential for surface-mediated interaction with protein surface, this suggesting their ability to disrupt enzymatic functions critical for fungal growth and pathogenicity. ZnO-NPs formed conventional hydrogen bonds with argenin (ARG) residues, and appear an unfeasible pump with LYS residue, suggesting a binding potential of ZnO-NPs to the protein’s surface site and may be leading to an allosteric or steric inhibition mechanism that changes the enzyme’s shape, which in turn alters or blocks the active site and prevents the substrate from binding. These preliminary interactions may interfere with protein function and contribute to the nanoparticles’ antifungal activity (Alikarami et al., 2025). Additionally, biostimulants and ZnO-NPs showed antifungal activity in agreement with previous researches by Zhang, Li (Zhang et al., 2019); Wei, Bi (Wei et al., 2020) which demonstrated that downregulation or mutation of CYP51 reduces ergosterol levels, compromises membrane integrity, and leads to growth inhibition in fungi such as *Fusarium sambucinum* and other pathogenic fungi. The integration of molecular docking with experimental antifungal assays strengthens the hypothesis that these compounds act through direct molecular inhibition, offering a sustainable and targeted approach for managing Fusarium wilt in tomato plants.

Seventy days of post-inoculation, untreated tomato plants exhibited shorter roots, reduced plant height, smaller leaf areas, lower fresh and dry weights, and yellowing leaves. Although not always statistically significant, treatment with ZnO-NPs, SA, and HA improved these growth parameters compared to the positive control plants. Plants that were inoculated typically exhibited a decrease in the number of fruits, leaves, and branches compared with the absolute control group. The browning and yellowing of branches and leaves were more evident in inoculated plants than in those that received the treatment. The application of HA at 100 mg/L led to the highest fruit production, whereas ZnO-NPs at 500 mg/L resulted in the highest number of branches. In contrast, the use of 0.5 mM SA had the least impact, with this group showing the most browning spots and yellowed leaves.

The positive ZnO-NPs impacts on plant growth can be ascribed to the essential role of zinc as a micronutrient, which is critical for processes such as cell elongation, division, and regulation of hormones, including auxin, gibberellins, and cytokinin biosynthesis. Furthermore, Zn enhances the activity of antioxidant enzymes, thereby aiding the defense of plants against pathogens (Kriti et al., 2020; González-Merino et al., 2021). Corroborating these observations, González‐Merino, Hernández‐Juárez (González-Merino et al., 2021) concluded that 1500 ppm ZnO-NPs significantly enhanced plant growth and reduced disease severity compared to inoculated controls. These findings underscore the potential of ZnO-NPs to promote plant health and mitigate pathogen damage.

Several studies have demonstrated significant effects of SA and HA against FW disease. El-Shennawy M.Z and Abd El-All (El-Shennawy and Abd El-All, 2018) reported that SA (30 mg/L) achieved the greatest reduction in disease incidence (75.85% and 76.26%) and severity (60.97% and 63.04%) during two tomato growing seasons. Khalil (2021) observed that combining *Trichoderma viride* with SA significantly reduced disease severity and incidence under greenhouse conditions. Similarly, HA has been found to effectively combat this disease. Abdel-Monaim, Ismail (Abdel-Monaim et al., 2011), Abdel-Monaim, Abdel-Gaid (Abdel-Monaim et al., 2012) showed HA significantly decreased disease incidence, improved plant height, and increased productivity (pods/plant, seed yield/hectare), alongside enhancing survival rates, fresh and dry weights in treated seedlings compared to untreated controls.

Biochemical analyses revealed notable increases in photosynthetic pigments and related parameters under different treatments. For instance, plants treated with ZnO-NPs (500 mg/L) had the highest pigment content, while ZnO-NPs (250 mg/L) maximized carbohydrate levels. The protein content was highest with SA (0.6 mM) and lowest with HA (150 mg/L). Parveen and Siddiqui (2021) highlighted the ability of ZnO-NPs to enhance the growth and photosynthetic pigments of tomatoes. Thunugunta, Channa Reddy (Thunugunta et al., 2018) and Abdelaziz, Salem (Abdelaziz et al., 2022) further emphasized the role of ZnO-NPs in boosting antioxidant enzyme activity and photosynthetic pigment content in eggplants. These results are consistent with those of previous studies (Abdel Latef et al., 2016; El-Shennawy and Abd El-All, 2018; Bala et al., 2019) and (El-Sheekh et al., 2020) which consistently demonstrated that SA, HA, and ZnO-NPs significantly enhance plant chemical composition and photosynthetic pigments in Fusarium-infected plants.

All treatments significantly enhanced antioxidant activity compared to both the absolute and Fusarium-inoculated controls, as evidenced by an increase in the total phenolic content, antioxidant capacity (TAC), and DPPH radical inhibition. The highest total phenolic levels were recorded with 0.6 mM SA, whereas the lowest was observed with 150 mg/L HA. The TAC was maximized in the inoculated control, whereas the negative control showed the lowest values. Notably, DPPH scavenging activity demonstrated an inverse trend, with 0.6 mM SA exhibiting the greatest antioxidant activity. Enhanced antioxidant capacity and phenolic content have been implicated in antifungal activity against *F. oxysporum* through mechanisms such as cell rupture, reduced ATP production, release of intracellular components, and chelation of iron ions, leading to oxidative damage (Maringgal et al., 2019). Exposure to ZnO-NPs significantly augmented the total antioxidant capacity (TAC), phenolic content, and other protective factors (Malik and Singh, 1980; Abdelaziz et al., 2022). SA exerts a protective effect on plants by inducing gene expression, regulating antioxidants and reactive oxygen species (ROS), and enhancing element absorption. It also enhances both enzymatic and non-enzymatic antioxidant systems to mitigate stress effects, including metal toxicity (Wang and Li, 2006; Moustafa-Farag et al., 2020). For example, El-Esawi, Elansary (El-Esawi et al., 2017) observed an increased antioxidant capacity in SA-treated rosemary under salinity stress. Similar studies have underscored the role of SA in fortifying defense mechanisms against FW (Bawa et al., 2019; Zhu et al., 2022). HA treatment consistently elevated the levels of ascorbic acid, superoxide dismutase, β-carotene, and α-tocopherol, which are vital for plant development and stress resistance (Abdel-Monaim et al., 2011). Further research indicated that HA treatments enhanced antioxidant capacity, including anthocyanins, phenols, and flavonoids, even under stress conditions (Abdel-Monaim et al., 2012; Akladious and Mohamed, 2018).

The present study evaluated the antifungal potential of salicylic acid (SA), humic acid (HA), and zinc oxide nanoparticles (ZnO-NPs) against Fusarium oxysporum and their impact on tomato plant growth and physiology. Our findings demonstrated that higher concentrations of ZnO-NPs significantly inhibited mycelial growth *in vitro* and improved plant performance under greenhouse conditions, consistent with previous reports on nanoparticle-mediated disease suppression (Servin et al., 2015; Dimkpa and Bindraban, 2018).

To address concerns regarding nanoparticle safety, NP-only treatments (without pathogen inoculation) were included in the greenhouse experiment. No symptoms of leaf burn, root damage, or yield penalty were observed at the tested concentrations (100–500 mg/L ZnO-NPs). These results suggest that ZnO-NPs, when applied within this range, are not phytotoxic and may even enhance plant vigor, aligning with earlier studies on ZnO-NPs as micronutrient sources (Mukherjee et al., 2016).

The environmental fate of ZnO-NPs is an important consideration for sustainable application. ZnO-NPs can undergo dissolution, aggregation, or transformation in soil, influencing their bioavailability and potential ecotoxicity (Dimkpa et al., 2020). While Zn is an essential micronutrient, excessive accumulation may pose risks to soil microbiota and groundwater. Therefore, future studies should investigate nanoparticle persistence, mobility, and interaction with organic matter under field conditions to ensure safe long-term use.

Current agricultural regulations in many regions mandate strict evaluation of nanoparticle formulations for toxicity, persistence, and bioaccumulation before approval for field use. Furthermore, the long-term impact on soil health, particularly on microbial diversity and functionality, remains a critical concern. Future studies should therefore focus on cost-benefit analyses, risk assessment, and comprehensive soil microbiome evaluations under realistic agronomic conditions to ensure sustainable and responsible deployment of nanotechnology in agriculture.

## 5 Conclusions

This study demonstrates the promising antifungal potential of environmentally sustainable compounds—zinc oxide nanoparticles (ZnO-NPs), salicylic acid (SA), and humic acid (HA)—against *Fusarium oxysporum*, the causative agent of *Fusarium* wilt in tomato plants. Molecular docking and experimental assays revealed that these agents interact directly with key fungal proteins, particularly sterol 14α-demethylase (CYP51), disrupting essential biosynthetic pathways. Among the tested compounds, SA exhibited the highest binding affinity and additional inhibitory effects on fungal signaling pathways, positioning it as a leading candidate for further development. Despite these advancements, future studies should explore the synergistic effects of combining SA, HA, and ZnO-NPs, optimize nanoparticle delivery systems, and investigate their interactions with the soil microbiome. Further *in vitro* dose–response experiments will be conducted to accurately determine the half-maximal inhibitory concentration (IC_50_) and confirm physiological relevance. Additionally, more advanced computational approaches, such as QM/MM simulations, will be employed to provide deeper mechanistic insights. Additionally, extensive field trials are needed to validate laboratory findings and assess long-term efficacy. Integrating these compounds into broader pest management strategies, alongside the development of resistant tomato cultivars and rapid detection systems, will be essential for sustainable and effective control of *Fusarium* wilt.

## Data Availability

The datasets presented in this study can be found in online repositories. The names of the repository/repositories and accession number(s) can be found in the article/**Supplementary Material**.

## Supplementary material

Supplementary Figure 1 **(A)** UV-vis spectrum of ZnO NPs (190–700 nm). **(B)** TEM micrographs showing round ZnO NPs. **(C)** FTIR spectrum displaying functional groups and structure of ZnO NPs.

Supplementary Figure 2 *In vitro* anti-fungal activity on *F. oxysporum* by different concentrations of ZnO-NPs and growth regulators after 9 days of incubation. The inhibition of mycelial growth according to **(A)** ZnO-NPs; **(B)** Salicylic acid, and **(C)** Humic acid concentrations.

Supplementary Figure 3 The effect of different treatments on morphology of 70-day-old *S. lycopersicum* plants. TO-: negative control; TO+: positive control; SA= Salicylic acid; HA= humic acid; ZnO NPs= Zinc oxide nanoparticles.
