# Supplementary material for: Eco-friendly control of fusarium wilt in tomato: molecular docking and functional analysis of ZnO nanoparticles and biostimulants
Source: Front Plant Sci. 2025 Dec 9;16:1687653. doi: 10.3389/fpls.2025.1687653 (PMC12722457; doi:10.3389/fpls.2025.1687653)
Supplement: Supplementary Figure 1 — (A) UV-vis spectrum of ZnO NPs (190–700 nm). (B) TEM micrographs showing round ZnO NPs. (C) FTIR spectrum displaying functional groups and structure of ZnO NPs. [file DataSheet1.docx]

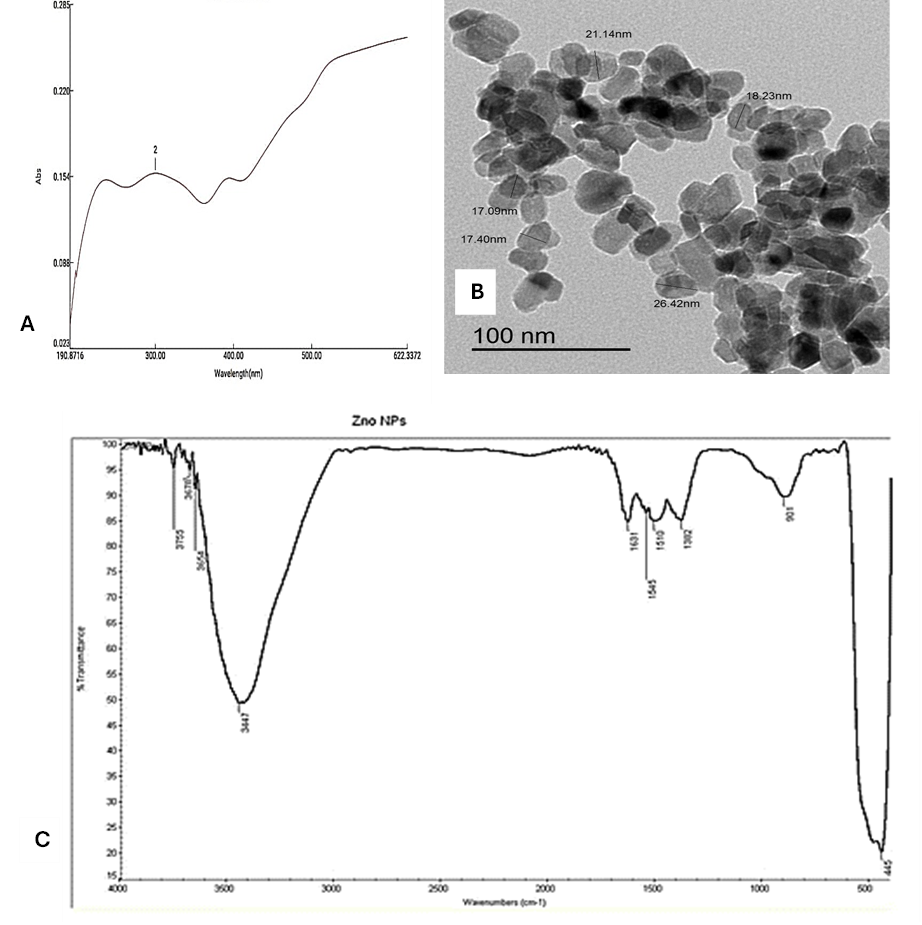


**Figure S1**. **A)** UV-vis spectrum of ZnO NPs (190–700 nm). **B)** TEM micrographs showing round ZnO NPs. **C)** FTIR spectrum displaying functional groups and structure of ZnO NPs.


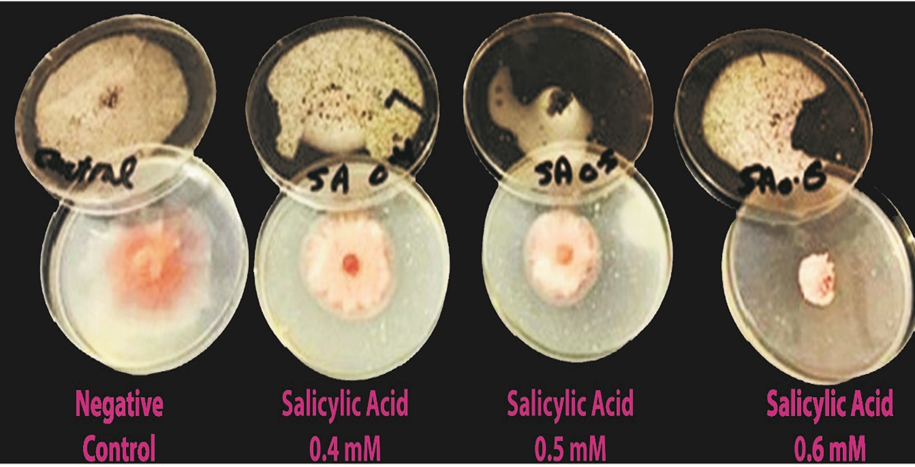


**B**


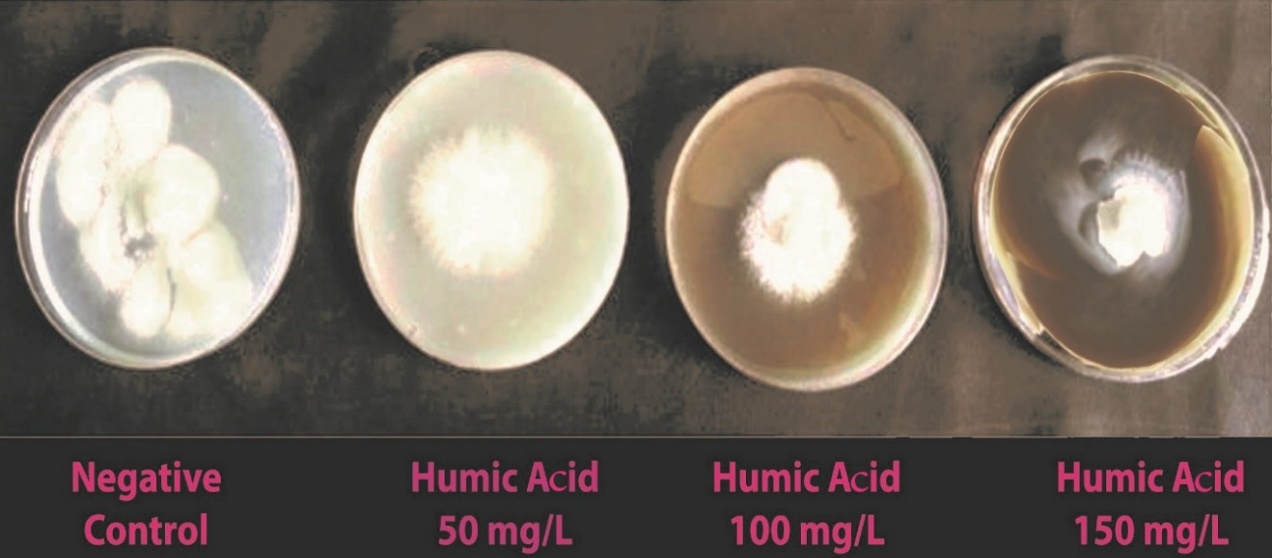


**C**


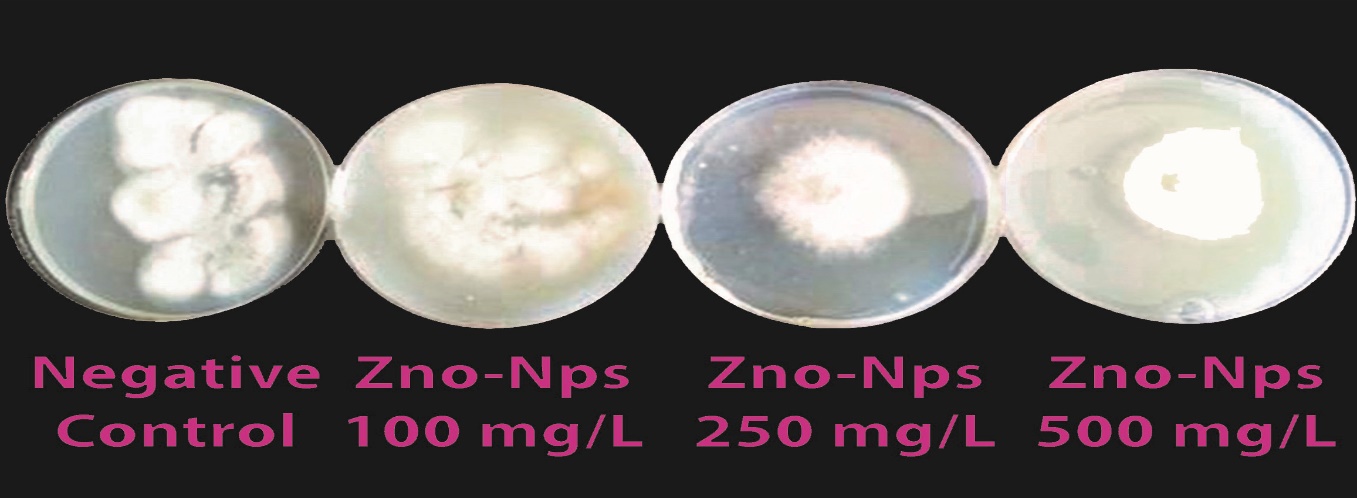


**A**

**Figure S2.** *In vitro* anti-fungal activity on *F.* *oxysporum* by different concentrations of ZnO-NPs and growth regulators after 9 days of incubation. The inhibition of mycelial growth according to A) ZnO-NPs; B) Salicylic acid, and C) Humic acid concentrations.


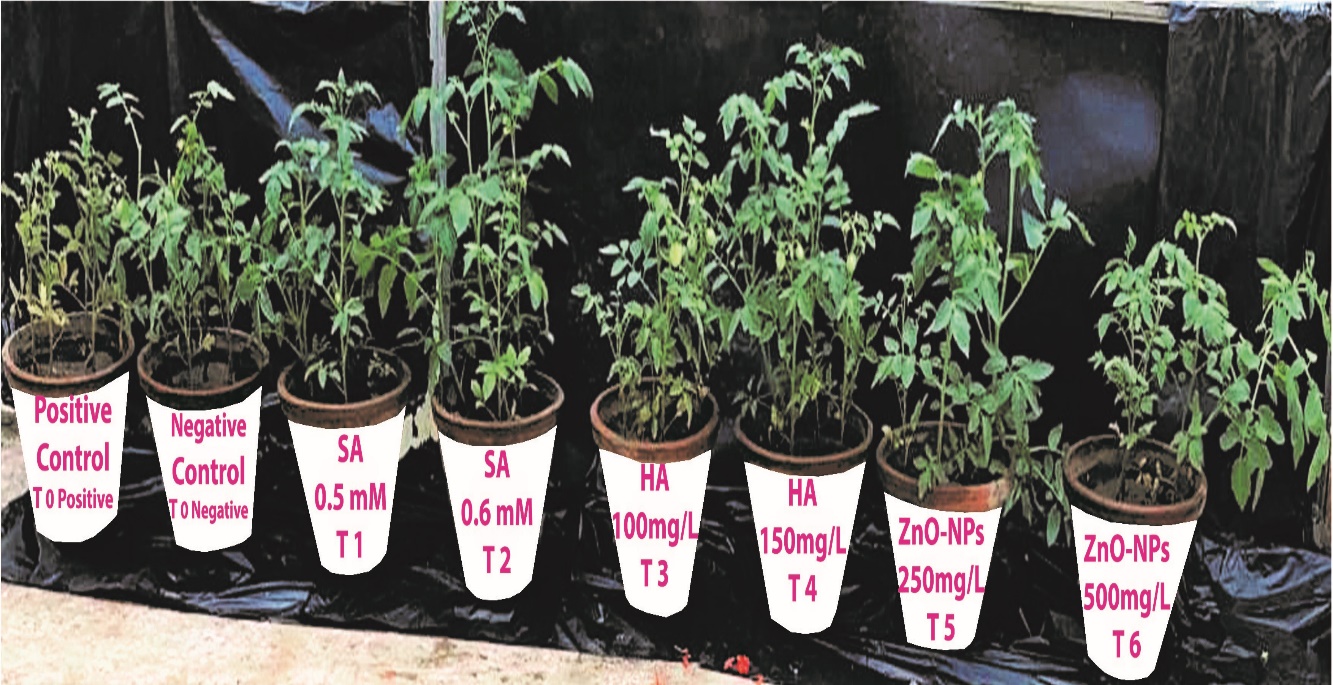


**Figure S3.** The effect of different treatments on the morphology of 70-day-old *S.* *lycopersicum* plants. TO-: negative control; TO+: positive control; SA= Salicylic acid; HA= humic acid; ZnO NPs= Zinc oxide nanoparticles.
